# Calculating the statistical significance of rare variants causal for Mendelian and complex disorders

**DOI:** 10.1186/s12920-018-0371-9

**Published:** 2018-06-13

**Authors:** Aliz R. Rao, Stanley F. Nelson

**Affiliations:** 1 Department of Human Genetics, University of California, Los Angeles, California, Los Angeles USA; 2 Department of Psychiatry and Biobehavioral Sciences at the David Geffen School of Medicine, University of California, Los Angeles, California, Los Angeles USA; 3 Department of Pathology and Laboratory Medicine, University of California, Los Angeles, California, Los Angeles USA

**Keywords:** SORVA, Intolerance, Genes, Protein domains, Mutational burden, Significance, Pathogenic

## Abstract

**Background:**

With the expanding use of next-gen sequencing (NGS) to diagnose the thousands of rare Mendelian genetic diseases, it is critical to be able to interpret individual DNA variation. To calculate the significance of finding a rare protein-altering variant in a given gene, one must know the frequency of seeing a variant in the general population that is at least as damaging as the variant in question.

**Methods:**

We developed a general method to better interpret the likelihood that a rare variant is disease causing if observed in a given gene or genic region mapping to a described protein domain, using genome-wide information from a large control sample. Based on data from 2504 individuals in the 1000 Genomes Project dataset, we calculated the number of individuals who have a rare variant in a given gene for numerous filtering threshold scenarios, which may be used for calculating the significance of an observed rare variant being causal for disease. Additionally, we calculated mutational burden data on the number of individuals with rare variants in genic regions mapping to protein domains.

**Results:**

We describe methods to use the mutational burden data for calculating the significance of observing rare variants in a given proportion of sequenced individuals. We present SORVA, an implementation of these methods as a web tool, and we demonstrate application to 20 relevant but diverse next-gen sequencing studies. Specifically, we calculate the statistical significance of findings involving multi-family studies with rare Mendelian disease and a large-scale study of a complex disorder, autism spectrum disorder. If we use the frequency counts to rank genes based on intolerance for variation, the ranking correlates well with pLI scores derived from the Exome Aggregation Consortium (ExAC) dataset (ρ = 0.515), with the benefit that the scores are directly interpretable.

**Conclusions:**

We have presented a strategy that is useful for vetting candidate genes from NGS studies and allows researchers to calculate the significance of seeing a variant in a given gene or protein domain. This approach is an important step towards developing a quantitative, statistics-based approach for presenting clinical findings.

**Electronic supplementary material:**

The online version of this article (10.1186/s12920-018-0371-9) contains supplementary material, which is available to authorized users.

## Background

Whole-exome sequencing has enabled the identification of causal genes responsible for causing hundreds of rare, Mendelian disorders in just a few years; however, there remain hundreds, if not thousands, more to be uncovered. The genetic basis has been determined for 4803 of the rare diseases [1], whereas the number of disease phenotypes with a known or suspected Mendelian basis lies close to 6419 based on data in Online Mendelian Inheritance in Man (OMIM) [1]. Next-gen sequencing (NGS) studies are certain to uncover many disease-phenotype relationships in the near future, but for cases involving rare diseases with limited sample sizes, determining causality between phenotypes and novel genes, and distinguishing true pathogenic variants from rare benign variants remains a challenge. Often disease causality of a given rare variant is only clear when additional affected individuals with similar rare variants in the same gene are identified, which can take years to occur due to the rarity of these disorders. Thus, improvements in determining disease causality or likely pathogenicity would greatly enhance efforts to prioritize genes and gene variants for further molecular analysis, even if only a single affected individual was identified.

Variants identified through broad based NGS technologies are typically classified as pathogenic, likely pathogenic, variant of uncertain significance (VUS) or likely benign according to multiple criteria, largely based on prior knowledge about the specific variant. Novel variants are evaluated individually and placed into discrete categories if they meet complex combinations of criteria, which include thresholds for allele frequency, segregation, number of affected unrelated individuals, and known functional relevance [2, 3]. For example, a variant would be deemed pathogenic if the allele frequency threshold falls below a given threshold and the variant segregates with a disorder in at least two unrelated affected families, or if other criteria are met. In brief, variants are evaluated individually based on variant-specific annotations.

An additional source of information that would aid in variant prioritization would be a gene-specific annotation describing mutational burden in the overall population. To illustrate, consider a gene that has very few functional variants in the general population, and several unrelated patients were found to carry distinct protein-altering, rare missense or potential loss-of-function (LOF) variants in the given gene and within a highly conserved protein domain. Under a model for a rare Mendelian disorder caused by highly penetrant variants, we assume that common variants cannot be considered causal, and rare variants in genes intolerant of mutations are deemed highly suspicious of being causal for disease even if no other information is known about the variants. Therefore, knowing the population-wide mutational burden of a given gene for rare variants would be informative.

While there are gene-ranking methods based on other parameters [4], recently several gene-level ranking systems have emerged based on measures for intolerance to mutations in the general population. The Residual Variation Intolerance Score (RVIS) generates a score based on the frequencies of observed common functional coding variants compared to the total number of observed variants in the same gene or protein domain [5, 6]. A second ranking system, in addition to these parameters, also incorporates the frequency at which genes are found to be affected by rare, likely functional variants, and their findings suggest that disease associations to genes which frequently contain variants, termed as FLAGS, should be evaluated with extra caution [7]. Next, the Exome Aggregation Consortium (ExAC) dataset provides missense *Z* scores that describe the degree to which a gene is depleted of missense and LOF variants compared to expected values. They base expected values on the frequency of synonymous variants, and provides pLI scores that describe probabilities of a gene being LOF intolerant [8, 9]. Of these two metrics, pLI is less correlated with coding sequence length and outperforms the *Z* score as an intolerance metric [8]. Another method, EvoTol, combines genic intolerance with evolutionary conservation of whole protein sequences or their constituent protein domains to prioritize disease-causing genes, and extends the RVIS method by leveraging the information on protein sequence evolution to identify genes where the number of mutations that are likely to be damaging based on evolutionary protein information is higher than expected [10]. Although these methods may be useful in ranking genes and prioritizing variants in order to highlight those in genes that frequently contain variants, neither results in a score that is directly interpretable in order to calculate statistics about NGS findings and determine the significance of seeing a variant in a given number of affected individuals.

One tool that calculates a *P-*value of finding a true association through clinical exome sequencing, RD-Match [11], allows researchers to calculate the probability of finding phenotypically similar individuals who share variants in a gene through systems such as Matchmaker Exchange. The tool incorporates the probability of an individual having a rare, nonsynonymous variant in a gene by taking the sum of the allele frequencies of all rare (MAF < 0.1%) nonsynonymous variants annotated in ExAC [8]. With higher MAF thresholds and large population sizes, this is problematic because an individual may have multiple variants in a gene that frequently contains rare variation, causing one to overestimate the fraction of the population carrying rare variants in the gene, hence the fixed, low MAF threshold. Furthermore, this tool is applicable to studies in which the affected individuals are selected based on phenotype as well as the prior knowledge that they share rare variants in a given gene. Finally, RD-Match does not allow researchers to customize variant filtering thresholds according to the disease model with regards to minor allele frequency or predicted consequence such as LOF or missense variant.

Another method that calculates the significance of NGS findings, the Transmission And De novo Association test (TADA), is a Bayesian model that combines data from de novo mutations, inherited variants in families, and variants in cases and controls in a population [12]. This method has been used to identify risk-conferring genes in whole-exome sequencing studies of autism spectrum disorders and neurodevelopmental delay [13–15]. While TADA analysis has proven to be a critical first step in the development of quantitative methods to assess risk genes, it is restricted to integrating trio and case-control data and is unable to leverage information from larger pedigrees, and whether siblings or distantly related individuals share the variants observed in the proband. Also, it does not incorporate any information from large reference datasets, and therefore, it cannot be used for calculating the *P-*value of findings in smaller studies.

Here we describe a method, named SORVA for Significance Of Rare VAriants, for ranking genes based on mutational burden. In addition to incorporating information from variant allele frequencies, we use population-derived data to precompute an unbiased, easily interpretable score, which allows one to calculate the significance of observed and novel rare variants and their potential for being causal of disease. One may then answer the question: what is the probability of observing missense variants in three out of ten unrelated affected individuals, for example, given that only one in a thousand individuals in the general population carry a missense variant in the gene? Essentially, a model can be constructed to estimate the probability of drawing *n* unrelated families with similar biallelic genotypes by chance from the general population [16]. Conversely, if one has a large list of variants of unknown significance, the significance level may be useful in prioritizing variants within the same category of pathogenicity, and in improving the interpretation of variants in studies of Mendelian genetic disorders.

## Results

For calculating the significance of seeing variants within a gene when sequencing multiple individuals affected for a rare, presumably Mendelian disorder, we first calculated the frequency of observing a variant in each gene in an individual within the population by using a large control dataset and collapsing variants in each gene. Calculations are based on data from 2504 individuals in the 1000 Genomes Project phase 3 dataset, which includes targeted exome sequencing data (mean depth = 65.7×) from individuals from five “superpopulations” (European, African, East Asian, South Asian, and ad-mixed American) [17]. We repeated the analysis for variants filtered according to various minor allele frequency and protein consequence thresholds that researchers may use when filtering variants. First, we filtered out common variants that met various minor allele frequency (MAF) thresholds used in the literature and others: 5, 1, 0.5, 0.1 and 0.05%. We then filtered rare variants according to two scenarios before collapsing variants across genes: 1) we included all protein-altering variants, i.e. those that cause a nonsynonymous change in the protein transcript or have a potential loss-of-function (LOF) consequence, and 2) we filtered for LOF variants only, i.e. splice site, stop codon gain and frameshift variants.

Below, we present general findings in population and molecular genetics that can be gleaned from the dataset, and illustrate how the dataset can be used in multiple studies, as a control group to vet candidate genes and variants.

### Population differences

**Fig. 1:**
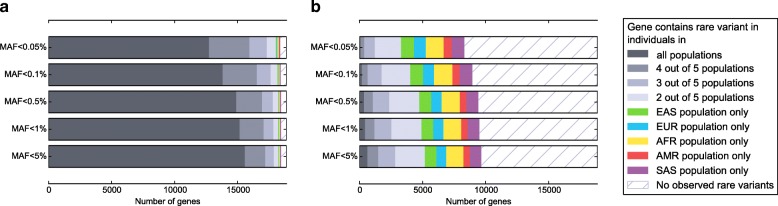
The proportion of genes (*n* = 18,877) containing rare variation in individuals in various populations. A gene was considered mutated if at least one individual was heterozygous or homozygous for an uncommon or rare (MAF < 5%) variant anywhere in the gene. Variants were filtered by predicted consequence for **(a)** protein-altering (missense or potential loss-of-function) variants, or **(b)** potential loss-of-function variants only. Abbreviations: EUR, European. AFR, African. EAS, East Asian. SAS, South Asian. AMR, ad-mixed American

Of 18,877 genes that are in the union of the Ensembl and RefSeq gene sets, most genes contained heterozygous or homozygous missense variants in individuals in all populations; only 2.3% contain no rare variants (MAF < 5%), and 1.0% of genes have an identified variant in only a single population. Lowering our MAF threshold does not decrease the number of genes much. Although, filtering variants to include only LOF variants reduces the number of genes containing variants in the dataset to 9641, or 51.1% of genes in the dataset. (Fig. 1) These results demonstrate that choosing the correct MAF threshold is not nearly as important as identifying the correct protein consequence threshold to use when filtering variants. For instance, including all missense variants when LOF variants are generally causal for a given disease would reduce power to detect the gene associated with the disease.

**Fig. 2:**
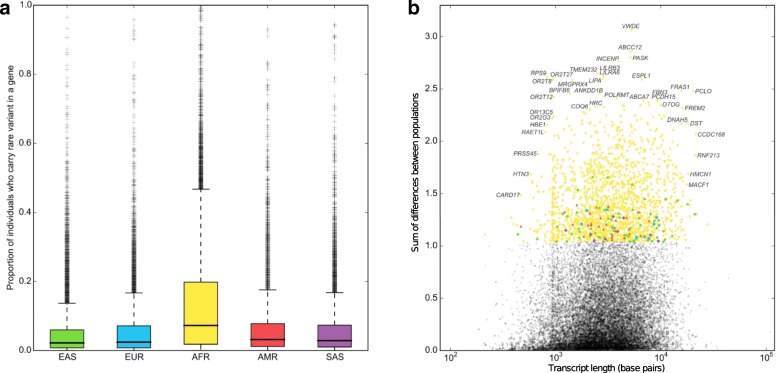
Population differences between the number of individuals mutated for a gene between populations. **a** Each data point in the histogram represents the proportion of individuals within a population who are heterozygous or homozygous for an uncommon (MAF < 5%) missense variant in a given gene. **b** The number of individuals carrying uncommon variants in a gene differs between populations. We plotted the variance of the count for each gene and colored high-variance genes to denote which population differed most from the mean

The number of individuals who carried a heterozygous or homozygous variant in a given gene was generally higher in the African population compared to other populations (Fig. 2a), which is expected given that African individuals are observed to have up to three times as many low-frequency variants as those of European or East Asian origin [17], which reflects ancestral bottlenecks in non-African populations [18]. Conversely, regarding genes for which the number of individuals with a rare variant in the gene differed between populations, the genes having the greatest difference between populations tended to diverge most in the African population. (Fig. 2b) Genes whose mutational burden diverges most between populations are significantly enriched for a large number of biological functional terms, including glycoprotein, olfactory transduction and sensory perception, cell adhesion, various repeats, basement membrane and extracellular matrix part, cadherin, microtubule motor activity, immunoglobulin and EGF-like domain. It is important to note differences between populations, because, in many cases, researchers would be advised to use control populations similar to their study population. However, if a gene is associated with a severe, childhood-onset disorder in one population, it is likely to be associated with disease in other populations, as well, and knowledge that a gene frequently contains variation in African populations would be useful in prioritizing candidate genes even if one is studying variation in another population. In this case, such information would point towards reduced likelihood for disease association.

### Properties of known disease genes

**Fig. 3:**
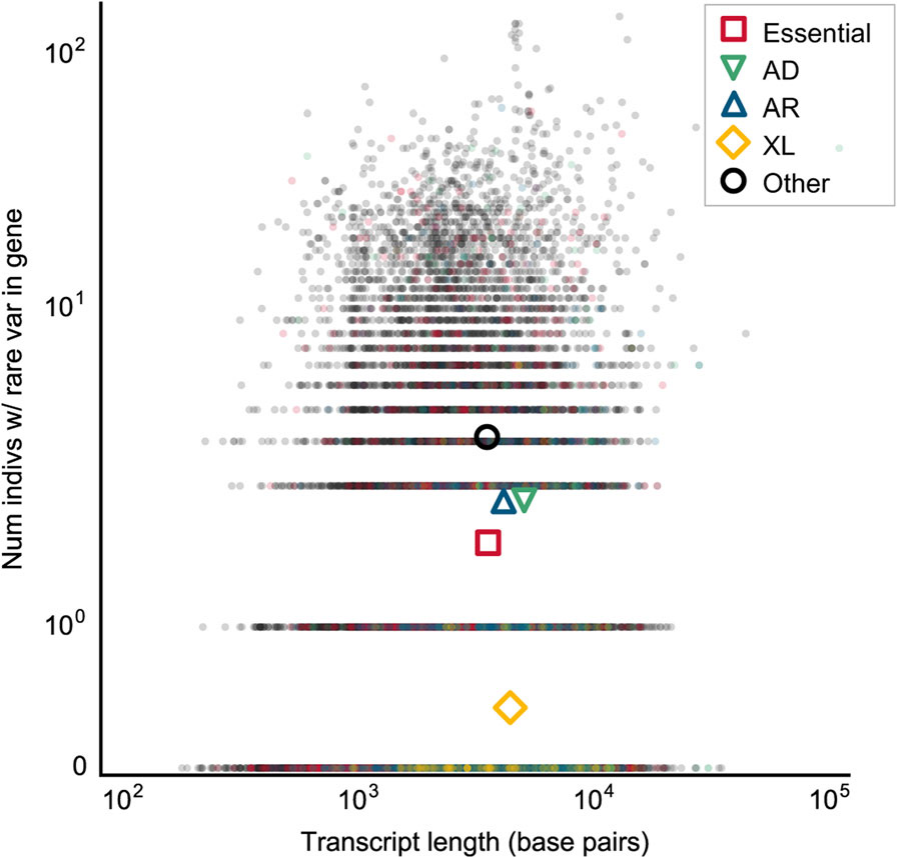
The number of individuals heterozygous for a rare (MAF < 0.5%) potentially LOF mutation in a gene. Each data point represents a single gene, mutated in the aggregate population (*n* = 2504 individuals). Genes are grouped according to whether they are an essential gene, or are known to cause autosomal dominant, autosomal recessive or X-linked disease. Colored shapes indicate the centroids of each group. Abbreviations: nonsyn, nonsynonymous. LOF, loss-of-function. AD, autosomal dominant. AR, autosomal recessive. XL, X-linked

To determine whether calculating the frequency of individuals who have a rare variant in a given gene in the general population may be helpful in determining which genes are more likely to cause disease, we compared the counts between multiple categories of genes: a) “essential” genes, defined as genes essential for cell survival in human cell lines, b) genes in which variants are known to cause autosomal dominant disorders, c) genes in which variants are known to cause autosomal recessive disorders, d) genes in which variants are known to cause X-linked disorders, and e) all other genes. As expected, fewer individuals carry rare, protein-altering or LOF variants in genes known to cause Mendelian disorders compared to other genes, and genes associated with X-linked disorders tend to be least tolerant of mutations (Fig. 3; Additional file 1). Although frequency counts overlapped between gene categories for every variant filtering threshold, clusters were most differentiated when plotting the proportion of individuals who are heterozygous for rare LOF variants in a gene. Furthermore, the differentiation between clusters increased as the MAF threshold became more stringent, as the datasets became enriched for deleterious variants that can only subsist at a low allele frequency in a population due to selective pressure. (Additional file 1).

Previous research suggests that 2.0% of adults of European ancestry and 1.1% of adults of African ancestry can be expected to have actionable highly penetrant pathogenic (including novel expected pathogenic) or likely pathogenic single-nucleotide variants (SNVs) in 112 medically actionable genes [2]. If we look for rare variants in 1000 Genomes Project individuals—benign as well as pathogenic variants—, we find that a larger proportion of 1000 Genomes Project individuals—5.8% of European individuals and 3.3% of African individuals—are heterozygous or homozygous for extremely rare (MAF < 0.0005) LOF variants in these 112 genes, highlighting the large number of benign variants that are found in the population at low allele frequencies and should be filtered out by manual curation.

### Depletion of variants in regions mapping to specific protein domains

It has been suggested previously that collapsing variants by protein domain could lead to improved gene-based intolerance scoring systems, as certain regions of the gene could be much more constrained than others [5]. We incorporated data for 322,772 protein domains from Interpro [19] and calculated the average number of individuals who have a variant in any given type of protein domain (Additional file 2), after filtering for rare (MAF < 0.5%), heterozygous LOF variants. Protein domains that are highly constrained, well covered during exome sequencing and rarely contain variants despite their large size include the Family A G protein-coupled receptor-like protein domain (Superfamily: SSF81321), which is found in 660 genes and has a mean length of 965 base pairs; none of the 2504 individuals carry rare variants in the region mapping to this protein domain. Other highly constrained protein domains that occur throughout the human genome include Glutamic acid-rich region profile (PfScan: PS50313), Proline-rich region profile (PfScan:PS50099), Immunoglobulin (Superfamily: SSF48726), and Cysteine-rich region profile (PfScan: PS50311). (Additional file 2) If an NGS study finds that affected individuals have rare variants in variation intolerant protein domains such as those listed, the variants would become highly suspicious of being causal.

**Fig. 4:**
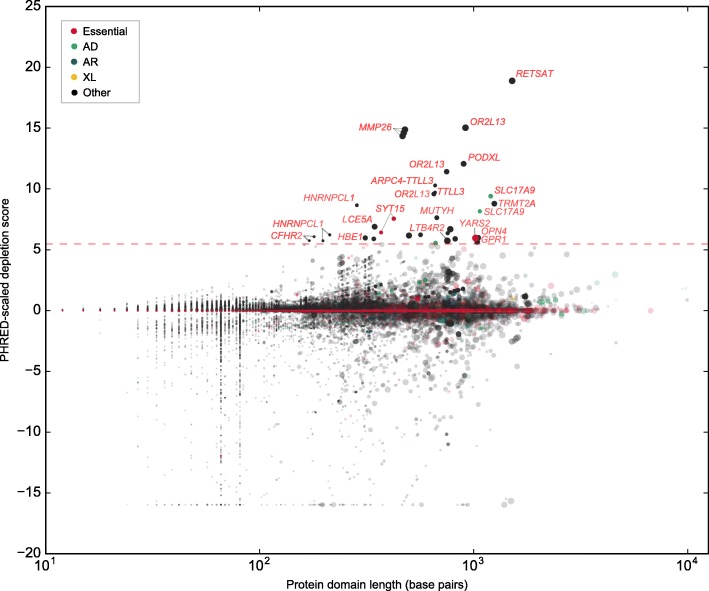
Depletion of rare, heterozygous LOF variants in regions mapping to protein domains. We plotted scaled protein domain depletion scores for each domain mapping within a gene; high scaled scores indicate that a protein domain is depleted of rare (MAF < 0.5%) mutations compared to the rest of the gene. Darkened points above the red dashed line represent protein domains that are significantly depleted of mutations after correcting for the number of genes remaining after filtering. Larger points indicate protein domains with a greater length in proportion to the transcript length. Points are colored if the protein domain is within a gene that is an essential human gene or is causal for a Mendelian disorder. Abbreviations: AD, autosomal dominant. AR, autosomal recessive. XL, X-linked

We also calculated whether specific genes contain protein domains that are significantly depleted of variation, given the frequency of variants in the gene overall. Filtering out protein domains in genes with no variants and those with missing information reduced the dataset to 67,138 protein domains in 7004 genes. 77 protein domains in 26 genes were significantly depleted of variation compared to the rest of the gene. Specifically, the number of rare (MAF < 0.5%), heterozygous LOF variants per individual in a protein domain were significantly lower than expected after correcting for multiple testing by the number of genes. (Fig. 4) Functional enrichment analysis in DAVID revealed that the most significant biological functions in the gene list were related to tubulin-tyrosine ligase activity (*P* = 0.015), and G-protein coupled receptor, rhodopsin-like superfamily (*P* = 0.05). Depletion values for all protein domains may be found in Additional file 3. Information about whether a protein domain is significantly depleted of variation despite being in a gene with frequently observed variation may be useful in distinguishing between pathogenic and benign rare variants within genes containing regions under different degrees of evolutionary constraint.

### Significance of findings in multi-family studies of rare genetic disorders

Below, we present methods for multiple study designs to calculate the significance of observing a given variant in a given gene. In the simplest case, a study involving a single family, calculating the *P*-value is relatively simple. Consider a case of a severe, pediatric-onset Mendelian disorder, in which both parents and the affected child are sequenced to identify the causal variant. If only de novo variants are identified within a putative gene, one can easily estimate the probability of at least one de novo mutation occurring in a gene by random chance; one could multiply the per-base mutation rate by the length of the gene transcript and make adjustments to account for CpG content related variation in mutation rates (Additional file 4).

In studies that identify both de novo and inherited variants in more complex family structures, calculating the significance of a variant is more complex. First, we generalize the equation for calculating the significance of observing a de novo mutation in a gene for studies involving multiple families. If multiple families are sequenced, the *P*-value of observing independent de novo events in the same gene in *s* out of *n* individuals is

$$ P=1- BinomCDF\left(s-1,n,{l}_{tx} dc\right) $$

where *l*_*tx*_ is the length of the transcript in nucleotide bases and *d* is the mean rate of de novo single-nucleotide variants (SNVs) arising per nucleotide per generation, *c* is the fraction of de novo events that meet our protein consequence threshold—2.85% if we consider only splice site altering or nonsense events, and 70.64% if we consider all protein-altering events, i.e. missense or LOF variants [20]—, and BinomCDF denotes the binomial cumulative distribution function. Consider the following example.

Clinical exome sequencing in four independent families identified de novo nonsense mutations in the gene *KAT6A* in all probands displaying significant developmental delay, microcephaly, and dysmorphism [21]. De novo nonsense mutations arising in this gene in all four individuals is highly unlikely by chance (*P =* 2.66 × 10^− 12^), and the statistical findings would support *KAT6A* as highly suspicious for causing the disorder. Further experiments and the identification of multiple other affected individuals by a separate study [22] confirmed this result.

If inherited variants are also observed in a gene, calculating the statistical significance of findings requires incorporating information about the number of individuals who carry a variant in the particular gene in the general population. The frequencies of the number of individuals who contain rare variants in a given gene or protein domain for various filtering thresholds may be queried through our online database called SORVA (https://sorva.genome.ucla.edu). (Additional file 5) Researchers can select the variant filtering thresholds identical to those used in hard filtering variants in a given study. Minor allele frequency thresholds range from 5%, useful for studies involving more common, complex disorders where less stringent filtering criteria are used, to 0.05% for studies involving extremely rare disorders. For genes that are rarely mutated, based on the expected number of individuals who carry a variant in the gene or protein domain in question, one can also calculate the significance of seeing the observed number of singletons (variants observed in a single independent individual), doubletons (variants observed in two individuals within a single family) or more complex cases as follows.

Let *f*_*hom*_ be the fraction of individuals in the general population with a homozygous variant in a gene or protein domain. Then, the *P*-value of seeing *k* individuals with a homozygous variant, out of *n* total unrelated affecteds is

$$ {P}_{k,n}=1- BinomCDF\left(k-1,n,{f}_{hom}\right) $$

where BinomCDF denotes the binomial cumulative distribution function.

If we sequence multiple individuals within a family, we can calculate the P-value of observing a given number of individuals with a variant in a gene under the following assumptions: 1) the gene rarely contains variants in the population, i.e. *f*_*hom*_ and *f*_*het*_ are small, and in this case, *f*_*hom*_ ≈ *f*_*het*_; and 2) the shared alleles within a family are shared identical-by-descent (IBD).

If we sequence full siblings, the *P*-value of seeing *k* sib pairs who share homozygous variants in a given gene, out of *n* total sib pairs is

$$ {P}_{k,n}=1- BinomCDF\left(k-1,n,\frac{1}{4}\ {f}_{hom}\right) $$

Another common scenario when sequencing individuals to determine the cause of an autosomal recessive disorder is to sequence distantly related affecteds in a pedigree with consanguineous marriages. In this case, the probability *P* that two sequenced individuals will share a homozygous can be calculated based on the pedigree structure and the corresponding path diagram, and the *P*-value becomes

$$ {P}_{k,n}=1- BinomCDF\left(k-1,n,{f}_{het}{\left(\frac{1}{2}\right)}^{E-1}\right) $$

where *E* is the number of independent edges in the paths connecting the two sequenced individuals through a single common ancestor. (Additional file 4).

If the affected individuals are heterozygous for the putative variants, the *P*-value is.

$$ {P}_{k,n,r}=1- BinomCDF\left(k-1,n,{rf}_{both}\right) $$

where *r* is the coefficient of relationship [23] or the fraction of the genome shared between affected family members, and *f*_*both*_ is the probability of an individual having either a heterozygous or homozygous variant in the gene of interest.

If multiple families and unrelated individuals had been sequenced with different degrees of relatedness, the *P*-value can be obtained by assuming that the control population is a pool of families with similar pedigree structures, calculating the probabilities of observing a combination of results that is at least as extreme as the current observation, and taking the sum of these probabilities.

To illustrate, consider that we have sequenced independent cases and sib pairs with a rare, autosomal dominant Mendelian disorder, and we observed that *k* of *n* independent cases (singletons) and *j* of *m* sib pairs (doubletons) have heterozygous variants in a given gene. We can calculate the probability *P*_*n,m,k,j*_ of observing exactly this number of successes based on the proportion of independent cases versus sib pairs and knowledge of the fraction of individuals heterozygous for rare variants in a given gene, *f*_*het*_. As an example, assume that we have sequenced two unrelated cases and four sib pairs concordant for disease status. After variant filtering, we note that three sib pairs and one unrelated case carry rare variants in the same gene. Then, we calculate the probability of observing any of the more extreme possible outcomes: observing 2 singletons and 3 sib pairs, 2 singletons and 4 sib pairs, or 2 singletons and 4 sib pairs who have heterozygous variants in the given gene. Then,

$$ P={P}_{\mathit{2,4,1,3}}+{P}_{\mathit{2,4,2,3}}+{P}_{\mathit{2,4,2,4}}+{P}_{\mathit{2,4,1,4}} $$

The formula for calculating *P*_*n,m,k,j*_ can be found in Methods, and detailed derivations of this and other equations can be found in Additional file 4. The a priori probability *p*, i.e. values for *f*_*het*_, *f*_*hom*_, and *f*_*both*_ for any given gene, can be queried from the SORVA dataset online, and standalone computer software for obtaining *p* and calculating *P*-values based on the methods described herein is also available on our website.

### Significance of findings in large-scale studies of complex disorders

**Fig. 5:**
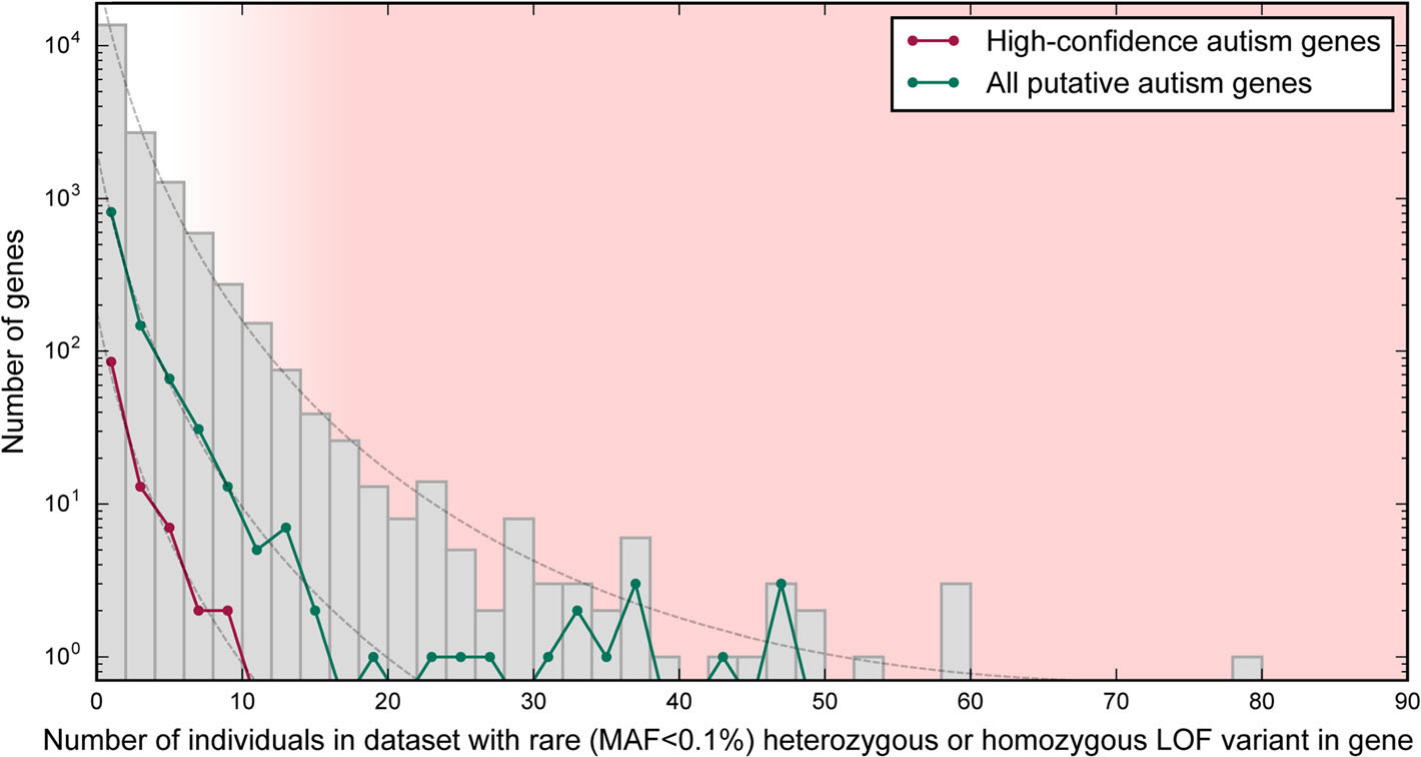
Histogram of the number of individuals with rare LOF variants in putative autism genes. The distribution of the number of individuals with a rare variant (MAF < 0.1%) in all genes is nearly identical to the distribution for putative autism genes (*N* = 1145) and high-confidence autism genes (*N* = 109) (dashed lines), suggesting that the genes may have been associated with autism by chance. Genes that frequently contain rare LOF variants in the population (red shaded region) are unlikely to be causal for ASD

In complex disorders where most of the genes contributing to risk remain unknown, our dataset may be used to provide additional evidence supporting novel gene findings and provides a simple method to calculate the significance of observing variants in a given gene in a large-scale study. As an example, several large-scale whole-exome sequencing (WES) studies have been carried out to-date in trios and quads to elucidate causal genes underlying autism spectrum disorders (ASD) [24–29]. However, genes identified as containing de novo variants rarely overlap between studies, raising the question of how many genes are truly causal and how likely genes are to be identified as associated with autism by chance in these studies as well as others. We assessed the number of individuals carrying rare (MAF < 0.1%), heterozygous LOF variants in 1145 genes cumulatively associated with ASD by more than a dozen studies, meta-analyses and reviews [14, 27, 30–47]. There was no significant difference between the distribution of values and that of all genes, and assuming that truly causal genes are more intolerant of rare LOF variants, our findings support the hypothesis that many genes could have been randomly associated with the disorder (Fig. 5, Additional file 6). Furthermore, there are 19 putative autism genes in which >0.5% of individuals carry rare, LOF variants. These genes are likely to be false positives, because no single gene contributes to a large proportion of autism cases. Our results highlight the need to perform statistical validation of findings involving genes associated with complex disorders.

Appropriately, several WES studies on ASD calculate the significance of their findings. For example, Sanders et al. demonstrate in a study which identifies de novo coding mutations in 928 individuals that finding two independent de novo mutations in a single gene is highly unlikely by chance, and this occurring is viewed as evidence for association between ASD and the gene *SCN2A* (sodium channel, voltage-gated, type II, α subunit) [28]. Neale et al. also consider the probability of seeing two independent de novo mutations in a single gene when evaluating their findings [25]. Iossifov et al. (2012) demonstrates that disrupted genes are significantly enriched for FRMP-associated function; however, they also highlight several individual non-FRMP-associated genes based on their plausibility to cause an ASD phenotype but make no attempt at applying statistics when considering these. In fact, de novo mutations in genes may have arisen in these genes by chance [24].

To validate our methods, we validated findings by O’Roak et al. (2012) [26], who reported de novo variants as well as inherited LOF variants in ASD cases. In a targeted sequencing study of 44 candidate genes in 2446 ASD probands, the authors found that six individual genes (*CHD8*, *GRIN2B*, *DYRK1A*, *PTEN*, *TBR1*, and *TBL1XR1*) had evidence of mutation burden for de novo variants, for which they calculated the *P*-value using simulations. Applying our methods, we find that more cases carry de novo variants than expected by chance in 6 out of the 6 genes. (Table 1). Furthermore, one additional gene, *ADNP*, was found to be significant using our method when only considering de novo variation in the cohort. One advantage to our method is that it allows one to incorporate information about inherited variants, and by doing so, 3 additional genes are found to contain more variation than expected by chance. For genes that rarely contain LOF variation in the population, observing more than one inherited variant in the cohort is unlikely to happen by chance, and the *P*-value decreases. To summarize, our methods approximate *P*-values obtained using more complex and computationally intensive methods such as simulations, with the advantage that it can incorporate information about both inherited and de novo variation, and the fact that it incorporates precomputed population level data makes our methods easy to apply to calculating the statistical significance of observing rare variants in a given gene.

**Table 1:** Validation of mutational burden findings in autism genes

	Number of indivs with variant				
Gene	LOF / de novo	Nonsyn / de novo	Inh / LOF	*P*	*P*_including inh vars_
*ADCY5*	0	2	1	0.723	0.446
*ADNP*	2	0	1	**1.1 × 10** ^**−3**^	**1.7 × 10** ^**− 3**^
***CHD8***	8	1	0	**1.7** × **10**^**−21**^	**1.7** × **10**^**− 21**^
***DYRK1A***	3	0	1	**5.9** × **10**^**−6**^	**1.1** × **10**^**−5**^
***GRIN2B***	3	1	0	**8.2** × **10**^**−5**^	**8.2** × **10**^**− 5**^
*LAMC3*	0	2	4	0.550	**0.022**
***PTEN***	1	2	1	**8.2 × 10** ^**−3**^	**9.2 × 10** ^**− 3**^
*SBF1*	0	2	1	0.833	0.502
*SETD2*	1	1	2	0.078	**0.0110**
*SGSM3*	0	2	2	0.086	**0.0471**
***TBL1XR1***	1	1	0	**0.0167**	**0.0167**
***TBR1***	2	1	0	**3.4 × 10** ^**−5**^	**3.4 × 10** ^**−5**^
*UBE3C*	0	2	1	0.432	0.291

In a targeted sequencing study of 44 candidate autism genes in 2446 individuals [26], 12 genes contained both recurring de novo variants and inherited LOF variants in multiple individuals, or had evidence of excess mutation burden of de novo variants. Gene names that are in bold were statistically significant in the original study. *P*-values calculated using our methods validate findings by O’Roak et al. [27] for all 6 of these genes, and one additional gene. If we also consider inherited LOF variants, 3 additional genes are statistically significant using our methods. Inherited nonsynonymous variants were not reported in the original study, hence the *P*-value is conservative. Abbreviations: *nonsyn* nonsynonymous variant or single amino acid deletion, *LOF* loss-of-function variant, *Inh* inherited

### Applications in predictive genomics

If a genetic disease is associated with the presence of variants in a given gene, information about the variants in the gene in affected individuals and in population controls can be used to more accurately assess the probability that a person will develop a disease given their genotype.

Consider a randomly chosen person from the general population who is undergoing prenatal genetic testing. Define *A* as the event that their child will be born with a disease, and *B* as the event that the child carries a rare, LOF variant in a given gene associated with the disease. For many heterogeneic Mendelian disorders, studies of large cohorts provide information regarding the relative contribution of individual causative genes and the genotype–phenotype correlations, giving us the conditional probability *P*(*B*|*A*). The term *P*(*A*) can be defined as the disease incidence, and the value of *P*(*B*), or the proportion of individuals carrying a rare, LOF variant in the gene, can be queried from our dataset. Then, according to Bayes’ theorem

$$ P\left(A|B\right)=\frac{P\left(B|A\right)\times P(A)}{P(B)} $$

we can calculate that the probability that the child will have the disorder. The following example illustrates such an application.

Consider that prenatal testing identified that a fetus is compound heterozygous for novel variants in the gene *POMGNT1*, which suggests a possible phenotype of congenital muscular dystrophy (CMD). It is known that 53% of patients with CMD have homozygous or compound heterozygous variants in one of six known CMD genes, 10% have homozygous or compound heterozygous variants in *POMGNT1*, and the incidence of CMD is estimated to be 1:21,500 [48, 49]. Since most mutations observed in affected individuals are novel and are not found in healthy population controls, we will assume a low MAF threshold of 0.1% for variant filtering. At this threshold, 2 out of 2504 individuals (0.08%) in our dataset have a rare protein-altering variant in the gene *POMGNT1*, therefore *P*(*B*) = 0.0008, and we calculate that the positive predictive value (PPV), the probability that the child will have the disease given a positive test result, is roughly 1.0%. Using this method, sensitivity, the probability *P*(*B*|*A*), is quite low (10%); whereas specificity is high (1-P(B) = 99.9%). If we aggregate data for all known CMD genes, we can increase sensitivity to 53% with a negligible decrease in specificity, due to the fact that the other CMD genes contains very few, in any variants in our dataset. This example highlights that sensitivity greatly depends on the proportion of cases that can be explained by variants in a given set of genes. This type of analysis thus has implications for interpretation of broad NGS-based prenatal testing and can be extrapolated as well to preconception testing and risk to potential children.

It is important to note that the extreme numbers involved—the very low prevalence of a disorder and in many cases, the fact that no individual on the 1000 Genomes Project dataset had been observed with variants in a gene, i.e. the lack of previous false-positive results—make it difficult to compute the PPV. A previous study suggests that the latter “zero numerator” problem can be solved using a Bayesian approach that incorporates a prior distribution describing the initial uncertainty about the false-positive rate [50]. Alternatively, the number of rare LOF variants observed in a gene has been published as part of the ExAC and GnomAD datasets, which contain information about 60,706 and 123,136 individuals, respectively [8]. Although only nonsense or splice site variants were included in the LOF classification, and they only include values for a single MAF threshold of 0.1%, the number can be used a rough estimate for *f.* Furthermore, if even the count obtained from GnomAD is zero, we can assume that *f* is less than 1/123136, or 3/123136 if we are being conservative.

To summarize, for monogenic disorders and disorders where there exist detailed phenotype-genotype correlation data, our dataset will provide the denominator in the equation to calculate the probability that an individual with a rare variant in a known disease gene will have a rare genetic disorder. As further research uncovers novel gene-disease associations, and as we increase the size of the public dataset from which *P*(*B*) values can be calculated, we can update expected false-positive rates and calculating PPVs will become increasingly accurate. As illustrated, our methods will be be useful for applications in predictive genomics, including prenatal testing and testing for late-onset genetic disorders.

### Comparison to other gene ranking methods

We applied our method to calculate the significance of several previous studies’ findings [51–77]. In all except one study where the Mendelian disorder was found to be caused by inherited disease variants (*N* = 20) [51–68, 76, 77], findings were confirmed to be significant using our methods, and in 11 out of 20 studies, *P*-values were highly significant (*P* < 0.0001). (Table 2, Additional file 7) In many studies, initial exome sequencing in a limited number of individuals is followed by sequencing of only the putative causal gene in a large number of individuals. In one such study, the *P*-value resulting from the initial exome sequencing is significant enough to suggest causality, and the follow-up sequencing essentially serves to establish the proportion of cases in which the phenotype is attributed to variants in the gene [61]. In others studies, however, the initial sequencing merely identifies potential candidate genes, and follow-up sequencing is required to achieve genome-wide significance [58, 59, 62, 76, 77]. In these cases, the second round of sequencing is not corrected for multiple testing, because only a single gene is interpreted during follow-up sequencing. (Additional file 7).

**Table 2:** Statistical significance of variants found to be causal in selected previous studies

Inheritance	Gene	Individuals sequenced	Indivs w/ var in gene	Variant consequence	Zygosity filter	MAF threshold^b^	f^a^	P-val	Approximate # of genes targeted	Corrected P-value
AR	*ISPD*	1 full-sibling doubleton and 5 unrelated affecteds	all	LOF	CHet/Hom	Exclusion	0	4.16E-008	7200	3.00E-004
AD	*CDKN1C*	1 third-cousin doubleton and 4 unrelated affecteds	all	nonsyn	Het	Exclusion	0.0044	5.24E-015	2500	1.31E-011
AD	*MLL2*	10 unrelated affecteds	7 / 10^c^	LOF	Het	Exclusion	0.0020	1.51E-017	20,000	3.02E-013
complex	*PBRM1*	7 unrelated affecteds	4 / 7^d^	LOF	Hom	none stated	0	5.56E-014	20,000	1.11E-009
complex	*WDR62*	2 affecteds in consanguineous family%	all	LOF	Hom	Exclusion	0	9.75E-008	20,000	1.95E-003
AR	*C5orf42*	6 unrelated affecteds	all^d^	nonsyn	Het	0.1%	0.0779	2.23E-007	20,000	4.46E-003
AR	*C5orf42*	2 affecteds in consanguineous family	all	nonsyn	Hom	none stated	0	4.88E-008	20,000	9.75E-004
complex	*AP4E1*	6 affecteds in a single, large family	all	nonsyn	Het	1%	0.0459	1.44E-003	530	0.76
AR	*ANTXR1*	2 unrelated affecteds from consanguineous families	all	nonsyn	Hom	0.1%	0	3.04E-013	144	4.38E-011
AR	*ITGB6*	2 unrelated affecteds	all	nonsyn	CHet/Hom	none stated	0.0012	1.44E-006	20,000	2.87E-002
AR	*CSPP1*	1 proband sequenced in region of homozygosity	all	LOF	Hom	1%	0	2.00E-004	40	7.99E-003
AR	*GMPPB*	3 unrelated affecteds and 3 sibs in consanguineous family sequenced in single candidate gene	all^d^	nonsyn	CHet/Hom	1%	0.0032	2.15E-014	1	2.15E-014
AR	*DHODH*	1 full-sibling doubleton and 2 unrelated affecteds	all	nonsyn	CHet/Hom	Exclusion	0.0004	1.35E-007	20,000	2.69E-003
AR	*SCN5A*	Gene screened in 10 affecteds from 7 families	5 / 10	nonsyn	CHet/Hom	none stated	0.0012	9.25E-010	1	9.25E-010
AD	*MAX*	3 unrelated affecteds	all	nonsyn	Het	Exclusion	0.0064	2.61E-007	20,000	5.22E-003
AR	*GPSM2*	1 proband sequenced in region of homozygosity	all	LOF	Hom	Exclusion	0.0000	2.00E-004	66	1.32E-002
AR	*TBC1D24*	15 unrelated affecteds	6 / 15	nonsyn	CHet/Hom	1.00%	0.0004	2.02E-017	20,000	4.05E-013
AR	*PGM3*	3 unrelated affecteds	all	nonsyn	CHet/Hom	0.3%	0.0004	6.37E-011	20,000	1.27E-006

Applying our methods to previous NGS findings, in which researchers filtered variants using various criteria, would have statistically validated findings in silico. See Additional file 7 for more details. ^a^The parameter *f* denotes the proportion of individuals in the 1000 Genomes Project dataset who have a rare variant at least as severe as the identified variants. ^**b**^We used a threshold of MAF < 0.1% for studies with no specific MAF threshold. A MAF threshold labeled exclusion refers to studies where variants were not filtered for a given threshold and variants were excluded based on their presence in public databases such as dbSNP. ^c^Follow-up Sanger sequencing identified mutations in 2 out of 3 exome-negative cases. ^d^Follow-up sequencing of the given gene identified further mutations in multiple additional cases. Abbreviations: *MAF* minor allele frequency, *AD* autosomal dominant, *AR* autosomal recessive, *XL* X-linked, *nonsyn* nonsynonymous variant, *LOF* loss-of-function variant, *Het* heterozygous, *Hom* homozygous, *CHet/Hom* compound heterozygous or homozygous

The rankings of frequencies at which a gene contains rare, deleterious variants is comparable to previously published gene ranking methods for prioritizing variants. The list of genes sorted and ranked according to the number of individuals carrying rare (MAF < 0.5%) heterozygous, loss-of-function variants correlates well with genes ranked based on pLI scores, which describe the probability that a gene is intolerant of LOF variation (ρ = 0.515) [8, 9]. These scores were derived from the ExAC dataset consisting of exome sequencing data from 60,706 individuals. The order in which ExAC pLI score ranks genes correlates more closely with SORVA rankings than rankings based on EvoTol [10] (ρ = 0.400), RVIS [5] (ρ = − 0.157) and FLAGS [7] (ρ = 0.278) methods.

We compare methods in their ability to prioritize disease-causing genes from the Online Mendelian Inheritance in Man (OMIM) database [1]. pLI scores, EvoTol, and RVIS outperform SORVA for known autosomal dominant disease genes, however all methods perform similarly for autosomal recessive genes, and SORVA outperforms EvoTol, RVIS, and FLAGS for genes known to cause X-linked disorders. (See Additional file 8 for receiver operating characteristic (ROC) curves.)

## Discussion

We demonstrate the utility of using mutational burden data to aid in prioritizing variants in silico and quantifying the significance of seeing a variant within a gene. We have shown this using examples from previous studies encompassing multiple NGS study designs and disease inheritance models. Other metrics such as gene constraint pLI scores and EvoTol rankings [9, 10] are appropriate for prioritizing genes by their likelihood of causing genetic disorders, but our methods will calculate the statistical significance of findings based on the constellation of families and individuals that variants are seen in, independent of how genes were prioritized initially.

Although there was some variation between the frequency of individuals with a rare variant in a given gene between populations, and selecting a comparable population to a study would be ideal when calculating variant significance, this restriction is not necessary. To illustrate, if individuals in the African population frequently carry LOF variants in a gene but this does not hold true for another population that more closely matches the study population, one may nevertheless consider the gene to be less likely to cause a rare Mendelian disorder.

A limitation of this method of ranking genes is that genes are prioritized on the basis of their likelihood of being involved in disease in general rather than in the specific disease of interest [4]. On the other hand, this can be viewed as a benefit in the sense that results are unbiased and do not depend on previously existing annotations, which would bias rankings to prefer known and well-studied genes. This bias is a known issue in the interpretation of clinical variants [78]. To illustrate, Bell et al. discovered that an unexpected proportion (27%) of literature-annotated disease variants in recessive disease-causing genes were incorrect [79], and Piton et al. estimated that 25% of X-linked intellectual disability genes are incorrect or require further review based on allele frequency estimates that have become more accurate with the availability of large-scale sequencing datasets [80]. Disease genes that are incorrectly annotated as disease-causing may explain the lack of difference between the average number of individuals carrying variants in genes causal for autosomal dominant and autosomal recessive genes. One would expect decreased counts for autosomal dominant disease genes due to stronger purifying selection among deleterious variants that arise in these genes, where a single variant may be sufficient to cause disease [81]. Another possibility is that the sample size may be too small to include a sufficient number of individuals who are carriers for rare, deleterious variants in recessive disease genes.

Future improvements to our methods would include increasing the amount of genetic information from unaffected individuals. Our results suggest that for most applications, low MAF thresholds should be used to achieve power to detect genes associated with disease; however, at thresholds of MAF < 0.0005, most genes will lack any data; e.g. there will be no individuals observed who are carriers of LOF variants. The SORVA dataset is useful in its current state with data from a relatively small number of individuals, but increasing the population size by several orders of magnitude will increase the utility of the application. The recently approved Precision Medicine Initiative will fund sequencing and data collection from 1 million or more Americans and make the data accessible to qualified researchers, and the methods described in this manuscript could be applied to this larger dataset and contribute towards the aim of this initiative to generate knowledge applicable to the whole range of health and disease [82].

Additional improvements would include incorporating additional information regarding specific categories of variants, such as the degree to which stop codon gain (also known as nonsense) variants in a gene are constrained to the end of the gene. Knowing whether an essential gene is highly intolerant of nonsense mutations in only certain regions of the gene would allow one to lower the priority of nonsense variants in regions tolerant of mutations when evaluating variants in silico. For example, Li et al. exclude stop-gain variants occurring in the terminal gene exon and those that do not affect all transcripts of a gene when evaluating deleterious LOF mutations in a large cohort of individuals [83]. The limitation to providing individual-level mutational burden counts at such a high level of granularity is that researchers will be restricted to following the same methods of filtering and annotating variants. This would be problematic because, by default, many commonly-used software pipelines do not annotate variants with the information about the proportion of transcript truncated [84–89]. Selecting variant filtering thresholds in SORVA that are identical to those used in one’s study is essential in having comparable data with which to calculate variant significance. For this reason, we also did not filter missense variants based on annotations from commonly tools such as SIFT [90], PolyPhen-2 [91], and CADD [92], which provide an interpretation of mutation impacts.

## Conclusions

Our methods provide a score for prioritizing variants within a gene that is unbiased and directly interpretable. Restricted by the sample size of our dataset, we provide limited population-level data, and adding more data will greatly improve the utility of our method. However, even in its current state, SORVA is useful for vetting candidate genes from NGS studies and allows researchers to calculate the significance of seeing a variant in a given gene or protein domain, which is an important step towards developing a quantitative, statistics-based approach for presenting clinical findings.

## Methods

### Datasets

Genomic data and allele frequencies for calculating a priori probabilities of observing a variant within a gene were obtained from the 1000 Genomes Project (phase 3 variant set) [17]. This variant set contains 2504 individuals from 26 populations in Africa (AFR), East Asia (EAS), Europe (EUR), South Asia (SAS), and the Americas (AMR).

### Bioinformatics pipeline

Genomic annotations were assigned to each variation using *SNP & Variation Suite (SVS)* v8.1 [84] with the following parameters: gene set Ensembl release 75 [93], human genome version GRCh37.p13. Variants were filtered for coding mutations that result in a change in the amino acid sequence (e.g. missense, nonsense and frameshift mutations), or mutations that reside within a splice site junction (intronic distance of 2 base pairs). Biallelic data was recoded based on an additive model to correct for MAF of variants on the X chromosome for male samples, using a script in SVS. Variants were then filtered for minor allele frequency thresholds of MAF < 5%, < 1%, < 0.5%, < 0.1% and < 0.05%, based on allelic frequency within the dataset. For each filtered list of variants, we collapsed variants by gene and performed the following two scenarios: 1) an individual was counted as having a rare variant in a gene if the variant mapped to any transcript of a gene; 2) we counted the number of variants in a given gene per individual, i.e. if an individual carried two rare mutations within a gene, they were counted twice. In a separate analysis, we collapsed variants by protein domains obtained from Interpro [19] using the Ensembl API [86]. Finally, we repeated each analysis using a subset of the 1000 Genomes Project data grouped according to superpopulation. Variant collapsing methods were performed using a custom Python script run by SVS, and an individual was counted as having a rare variant in a gene if the variant mapped to any transcript of a gene.

In addition to replicating the analysis for gene versus protein domain, for each population, and for each MAF threshold, we also repeated the calculations for multiple categories of predicted variant consequence on the protein transcript. The two categories were 1) nonsynonymous variants or those predicted to be more severe by Ensembl [93], briefly nonsynonymous or LOF variants, and 2) potential LOF variants (includes splice site, protein truncation stop codon gain mutations, and frameshift indels).

### Comparison of disease gene categories

To determine whether our results show concordance with studies identifying essential genes critical for the survival of a human, we compared the number of individuals with rare, deleterious mutations between gene lists containing essential human genes, those known to cause Mendelian diseases, and control genes, defined as genes not included in either category. We considered genes to be essential human genes if they were determined as such in at least one of the following two studies. The first essential human gene set is defined as ‘core’ essential genes that are required for fitness of cells from both the HAP1 and KBM7 cell lines, determined through extensive mutagenesis in near-haploid human cells (*N* = 1734) [94]. The second essential human gene set consists of genes essential to four screened cell lines, KBM7, K562, Raji and Jiyoye, determined using the CRISPR system. From the latter set, we selected genes with an adjusted *P*-value CRISPR score < 0.4025 for each cell line (*N* = 1878) [95].

To identify genes known to cause Mendelian disease, we parsed data from Online Mendelian Inheritance in Man (OMIM) [1] and identified phenotype descriptions with known molecular basis. We parsed the genotype description field for the gene name and the following phrases: ‘caused by heterozygous/homozygous mutation’, ‘autosomal recessive’, ‘autosomal dominant’, ‘X-linked’, ‘on chromosome X’, and categorized genes as autosomal recessive (AR) (*N* = 655), autosomal dominant (AD) (*N* = 785), and X-linked (XL) (*N* = 126) accordingly.

### Comparison of gene ranking methods

Genic mutational intolerance scores were obtained from four previous studies and included the Residual Variation Intolerance Score (RVIS) [5], scores from Shyr et al. 2014 (FLAGS) [7], pLI scores based on the ExAC dataset [8, 9], and EvoTol scores [10]. We considered 15,266 genes that were found in all four datasets, as well as ours, and ranked genes based on scores obtained using each method. Spearman’s rho test [96, 97] was used to measure the size and statistical significance of the association between the rankings obtained from ExAC and those obtained by RVIS, FLAGS and SORVA methods. This test measures the strength and direction of association between two ranked variables.

In order to assess the performances of all five methods when prioritizing putative disease genes and plot receiver operating characteristic (ROC) curves, we used the sets of OMIM genes described earlier. We filtered the OMIM gene sets to overlap the 15,266 genes that were scored by all five methods. Genes were ranked according to each metric and a count of the number of disease-causing genes that would be found at each percentile are reported. In order to show the baseline prediction, the result of randomly assigning a percentile to each gene is also shown. SORVA genes were ranked according to the number of 1000 Genomes Project individuals who were heterozygous or homozygous for rare (MAF < 0.005) LOF variants in a given gene, and ties between genes were resolved based on the number or individuals who have rare (MAF < 0.005) LOF or missense variants in a gene, and finally less rare (MAF < 0.05) LOF or missense variants.

### Calculating depletion of variants in protein domains

We performed two analyses: first, we calculated whether protein domains in a gene were depleted of variation compared to the rest of the gene, and second, we calculated whether there were any types of protein domains that were depleted of variation in general across the entire genome.

First, for each protein domain mapping within a gene, we calculated whether domains were depleted of variation compared to the rest of the gene. Depletion was calculated as: (number of variants per individual in protein domain / number of variants per individual in gene × length of protein domain / length of transcript). A value of 1 is expected by chance, and a small value indicates protein domains most intolerant towards mutations. We then calculated the *P*-value of obtaining such a depletion score using the binomial cumulative density function, under the assumption that each site is equally likely to be mutated. This *P*-value is then “PHRED-scaled” by expressing the rank in order of magnitude terms rather than the precise rank itself. High scaled scores indicate that a protein domain is depleted of rare (MAF < 0.5%) mutations compared to the rest of the gene, hence protein domains with high scores tend to be enriched for highly mutated genes. We filtered out genes with no observed mutations and protein domains that span more than 50% of the length of the transcript, resulting in 7828 genes remaining.

Next, we calculated whether there were any types of protein domains that were depleted of variation in general across the entire genome. We weighted each gene with instances of the protein domain equally. In other words, if a gene had multiple instances of a protein domain, we first calculated the mean number of heterozygous rare (MAF < =0.5%) LOF variants observed (in the entire dataset of 2504 individuals) in either protein domain within the gene. Next, we calculated the mean and variance of the means for each gene.

To determine whether a protein domain was well covered by sequencing, we calculated the mean coverage of an instance of a protein domain in the 1000 Genomes Project sample HG00096 [17]. We calculated depth of coverage from phase 3 exome alignment data using GATK and custom code, which is available at https://github.com/alizrrao/DepthOfCoveragePerInterval.

### Combining *P*-values when calculating significance of observing given variants in sequenced families

Let’s assume that we sequenced individuals in families with multiple family structures, e.g. we have sequenced independent cases and sib pairs with a rare, autosomal dominant Mendelian disorder, and we observed that *k* of *n* independent cases (singletons) and *j* of *m* sib pairs (doubletons) have heterozygous variants in a given gene. In the control population, the fraction of unrelated individuals heterozygous for a variant in the gene is *f*_*het*_ ≈ *f*_*both*_ = *r*_0_*f*_*both*_ for *f*_*het*_ <  < 1 where the relationship coefficient is *r*_0_ = 1, and the fraction of sib pairs who share heterozygous variants in the gene is *r*_1_*f*_*both*_ where the relationship coefficient is *r*_1_=½. Weighting these by the fraction of unrelated individuals and sib pairs, the total fraction of “familial units” that do not have or share the variant is

$$ F=1-\frac{n}{n+m}{r}_0{f}_{both}-\frac{m}{n+m}{r}_1{f}_{both} $$

which equals the probability of a failure in any given trial. The probability *P*_*n,m,k,j*_ of having *n* + *m* trials and observing exactly *k* singleton successes and *j* doubleton successes is equal to:

*P*_*n*, *m*, *k*, *j*_ = *P*(*X* = *n* + *m* − *k* − *j*) × *P*(*Y* = *k*)

where *X* is a binomial random variable with *n* + *m* trials and probability of success equal to *F*, and *Y* is a binomial random variable with *k* + *j* trials and probability of success equal to

$$ \frac{r_0n}{r_0n+{r}_1m} $$

Finally, to calculate the *P*-value for observing *k* of *n* independent cases and *j* of *m* sib pairs who have heterozygous variants in a given gene, we calculate the probability *P*_*n,m,k,j*_ of observing exactly *k* singleton successes and *j* doubleton successes or any combination of outcomes that is less likely, and sum these values.

$$ P- value=\sum \limits_{a=0}^n\sum \limits_{b=0}^m{P}_{n,m,a,b}\left[{P}_{n,m,a,b}\le {P}_{n,m,k,j}\right] $$

The *P*-value can be derived in a similar manner for various experimental designs, where multiple families with different pedigree structures are sequenced to identify heterozygous variants shared by affected cases or, in case of an autosomal recessive disorder, homozygous or potential compound heterozygous variants. Additional details can be found in Additional file 4: Supplementary Methods.

## Additional files

Additional file 1: Number of individuals carrying a rare variant in a gene under various filtering thresholds. Each data point represents a single gene which contains a variant in the aggregate population (*n* = 2504 individuals). Calculations were repeated using multiple variant filtering thresholds to determine the scenario that most differentiates between essential genes, those known to cause autosomal dominant, autosomal recessive or X-linked disease, and other genes. We varied filters for type of variant (‘LOF or missense’ or ‘LOF only’), zygosity (Het or Hom) and MAF threshold. Colored shapes indicate the centroids of each group of genes. Abbreviations: LOF, loss-of-function; nonsyn, nonsynonymous or LOF; het, heterozygous; hom, homozygous; ess, essential; AD, autosomal dominant; AR, autosomal recessive; XL, X-linked. (PDF 29608 kb)

Additional file 2: Mean number of individuals mutated for different types of protein domains. We calculated the mean number of individuals (out of 2504 individuals) who carried mutations in a given type of protein domains, averaging per gene. (XLS 2951 kb)

Additional file 3: Variant depletion scores for all protein domain in every gene. For each instance of a protein domain in a gene, we calculated variant depletion scores to identify regions within a gene that may be under differing degrees of evolutionary constraint. (XLS 28555 kb)

Additional file 4: Supplementary methods. Includes derivation of equations and math used for calculating the significance of finding rare variants in a given gene. (PDF 173 kb)

Additional file 5: Screenshot of an example query run on SORVA. Users can select variant filtering thresholds such as population, MAF cutoff, zygosity and whether to consider only LOF variants or missense variants, as well. Output includes the number of individuals who carry a rare variant in the gene and in any protein domain that maps to the gene. (PNG 129 kb)

Additional file 6: List of candidate autism genes. Genes listed were used to produce Fig. 5. (XLS 102 kb)

Additional file 7: Calculating *P*-values for findings from previous whole-exome or targeted sequencing studies. The parameter *f* denotes the proportion of individuals in the 1000 Genomes Project dataset who have a rare variant at least as severe as the identified variants. A MAF threshold labeled exclusion refers to studies that did not filter by a given threshold and excluded variants based on their presence in public databases such as dbSNP; in such cases, results were calculated using a MAF threshold of 0.1%. Abbreviations: MAF, minor allele frequency; AD, autosomal dominant; AR, autosomal recessive; XL, X-linked; nonsyn, nonsynonymous variant; LOF, loss-of-function variant; Het, heterozygous; Hom, homozygous; CHet/Hom, compound heterozygous or homozygous. (XLS 44 kb)

Additional file 8: ROC curves for the selection of known disease-causing genes from gene rankings. Comparison between gene ranking metrics from SORVA, FLAGS, ExAC pLI score, RVIS, and EvoTol using the OMIM database, showing the cumulative percentage plots for the residual scores for three OMIM gene lists. The OMIM gene categories are **(a)** autosomal dominant disease causing (*N* = 681), **(b)** autosomal recessive disease causing (*N* = 556), and **(c)** X-linked disease causing (*N* = 118). SORVA were based on the number of 1000 Genomes Project individuals who were heterozygous or homozygous for rare (MAF < 0.005) LOF variants in a given gene. Dashed lines indicate control. Abbreviations: ROC, Receiver Operating Characteristic; AUC, area under the curve, LOF, loss-of-function. (PDF 83 kb)

## Abbreviations

ASD: Autism spectrum disorder
BinomCDF: Binomial cumulative distribution function
CMD: Congenital muscular dystrophy
ExAC: Exome aggregation consortium
IBD: Identical-by-descent
LOF: Loss-of-function
MAF: Minor allele frequency
NGS: Next-generation sequencing
OMIM: Online mendelian inheritance in man
PPV: Positive predictive value
RVIS: Residual variation intolerance score
SNV: Single nucleotide variant
SORVA: Significance Of Rare VAriants
TADA: Transmission and de novo association test
VUS: Variant of uncertain significance
WES: Whole-exome sequencing
