# Supplementary figures and images for: Calculating the statistical significance of rare variants causal for Mendelian and complex disorders
Source: BMC Med Genomics. 2018 Jun 13;11:53. doi: 10.1186/s12920-018-0371-9 (PMC6001062; doi:10.1186/s12920-018-0371-9)

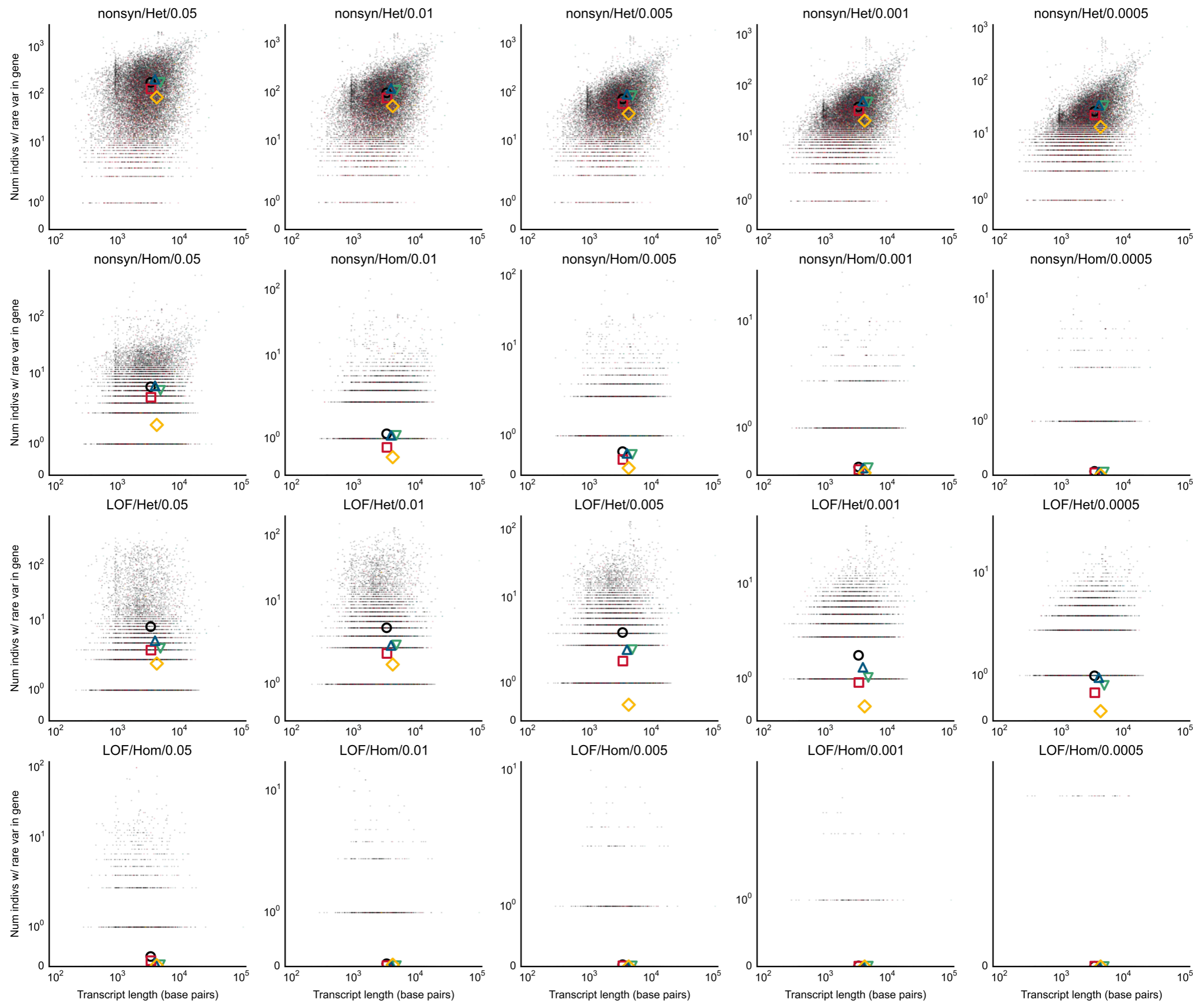

Supplement: Supplementary file 1 — Number of individuals carrying a rare variant in a gene under various filtering thresholds. Each data point represents a single gene which contains a variant in the aggregate population (n = 2504 individuals). Calculations were repeated using multiple variant filtering thresholds to determine the scenario that most differentiates between essential genes, those known to cause autosomal dominant, autosomal recessive or X-linked disease, and other genes. We varied filters for type of variant (‘LOF or missense’ or ‘LOF only’), zygosity (Het or Hom) and MAF threshold. Colored shapes indicate the centroids of each group of genes. Abbreviations: LOF, loss-of-function; nonsyn, nonsynonymous or LOF; het, heterozygous; hom, homozygous; ess, essential; AD, autosomal dominant; AR, autosomal recessive; XL, X-linked. (PDF 29608 kb) [file 12920_2018_371_MOESM1_ESM.pdf]

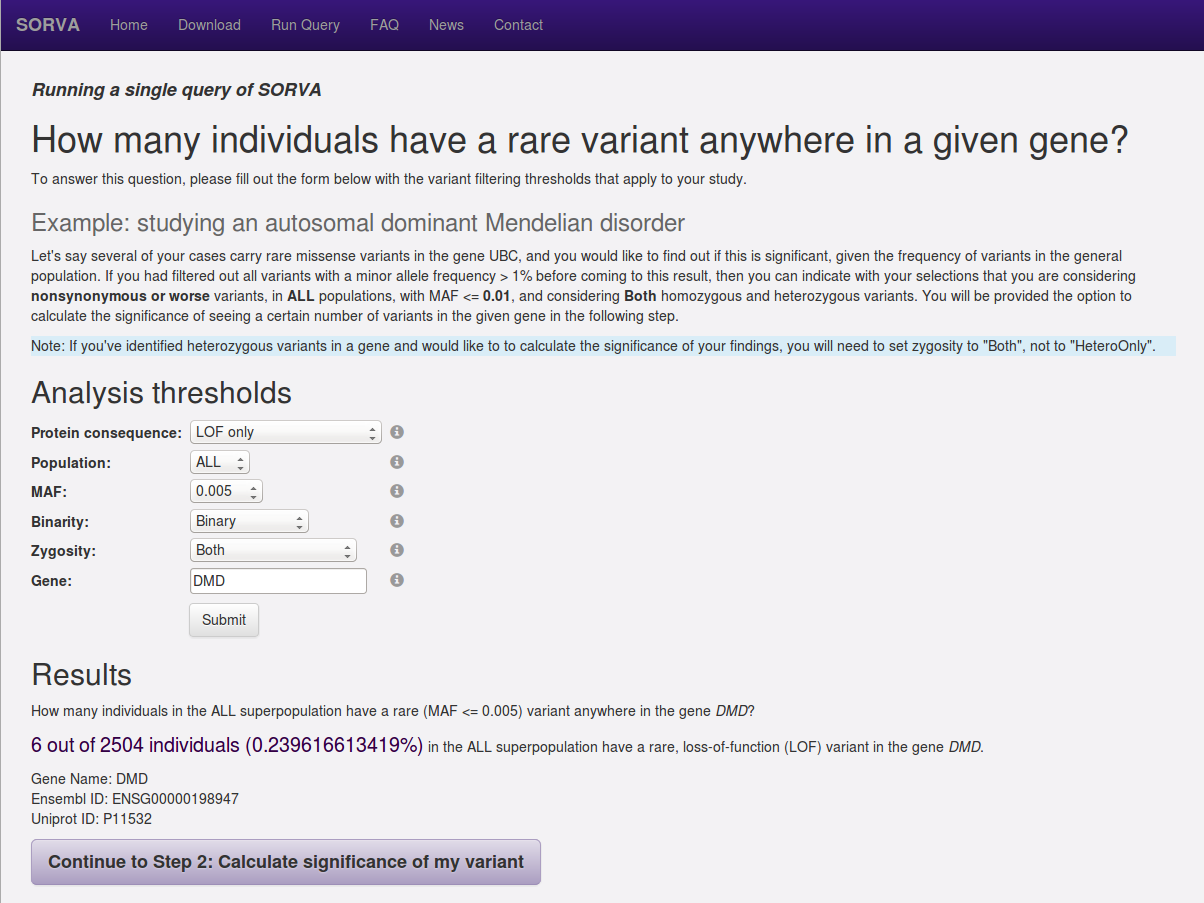

Supplement: Supplementary file 5 — Screenshot of an example query run on SORVA. Users can select variant filtering thresholds such as population, MAF cutoff, zygosity and whether to consider only LOF variants or missense variants, as well. Output includes the number of individuals who carry a rare variant in the gene and in any protein domain that maps to the gene. (PNG 129 kb) [file 12920_2018_371_MOESM5_ESM.png]

**a**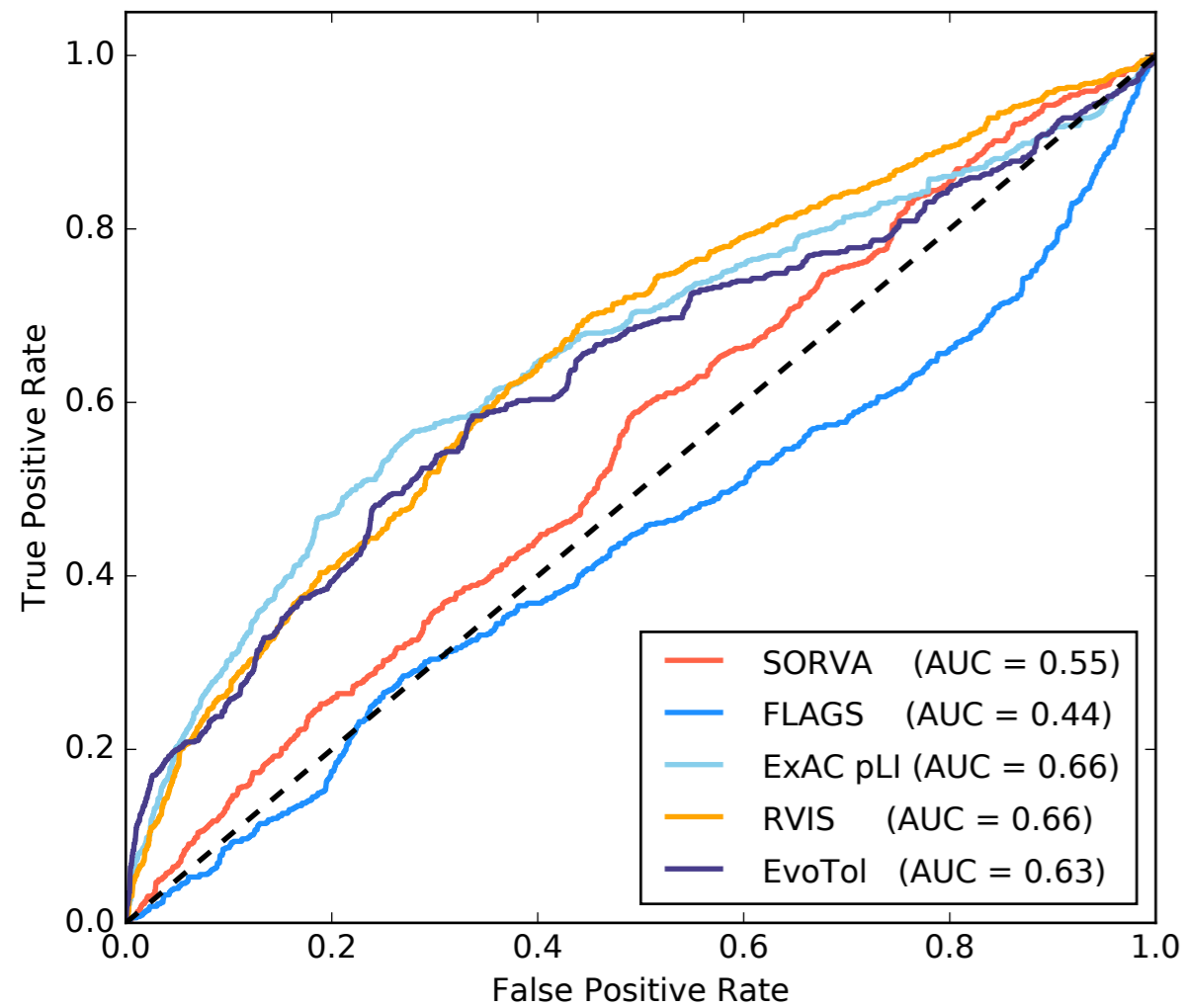**b**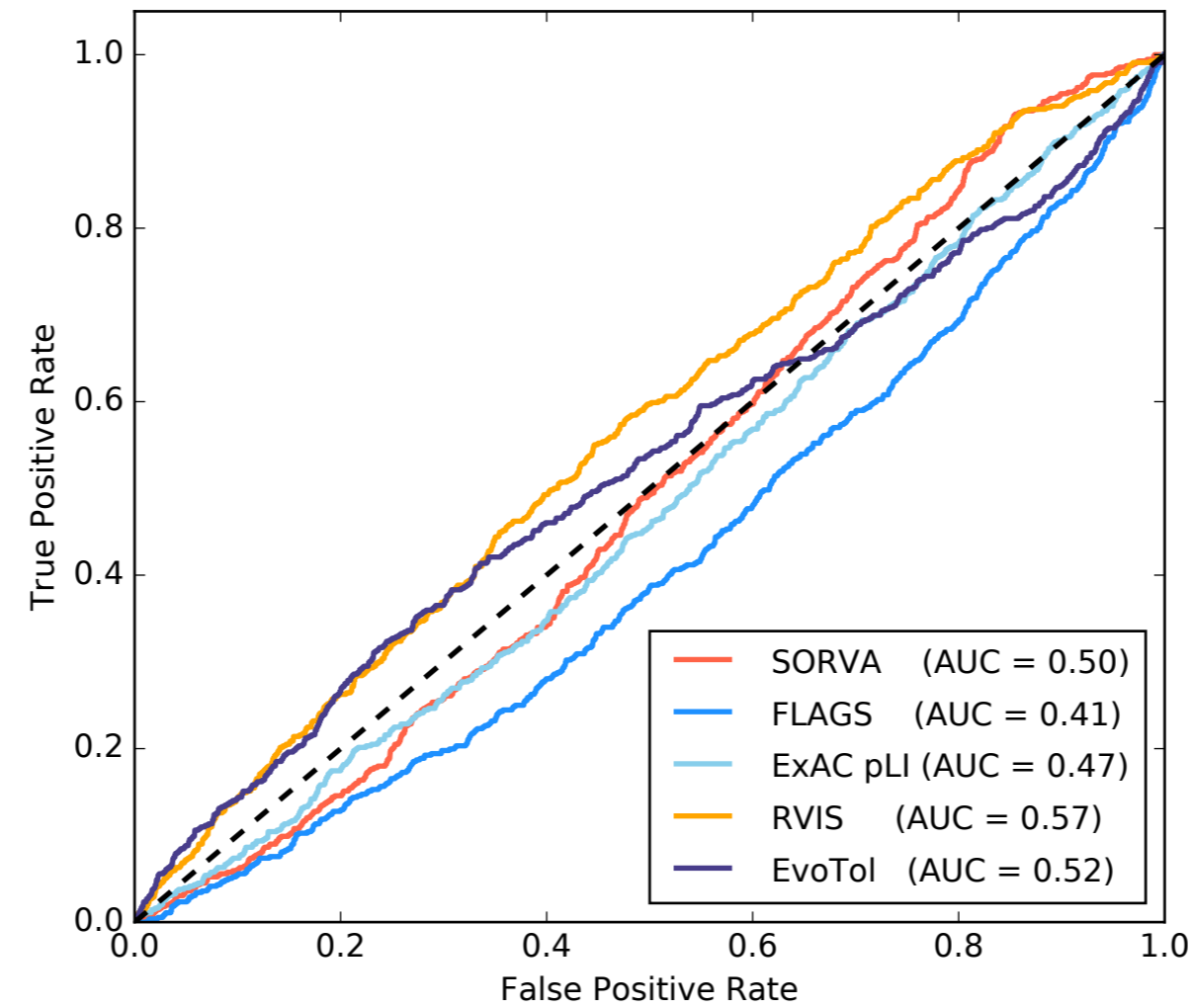**c**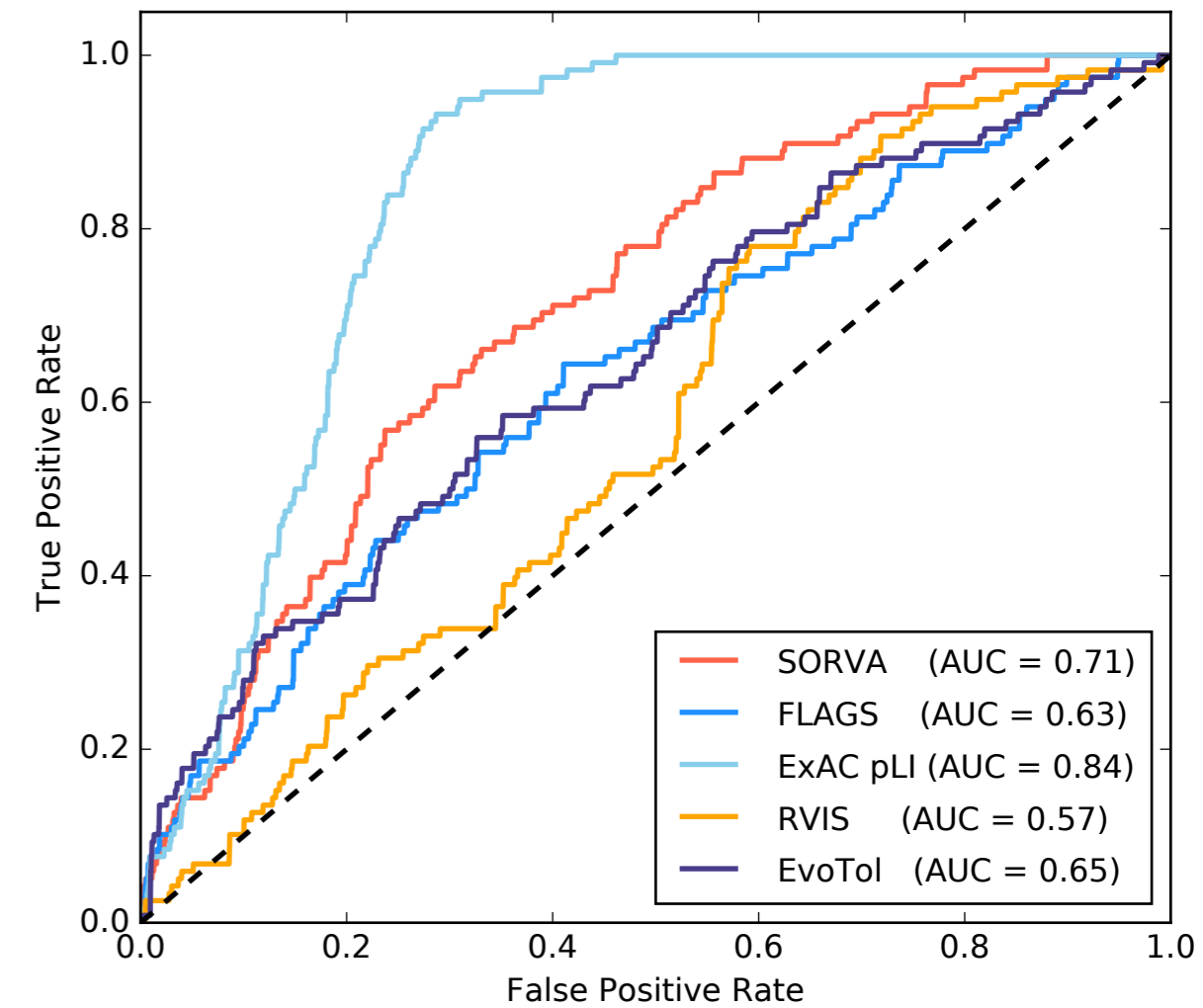

Supplement: Supplementary file 8 — ROC curves for the selection of known disease-causing genes from gene rankings. Comparison between gene ranking metrics from SORVA, FLAGS, ExAC pLI score, RVIS, and EvoTol using the OMIM database, showing the cumulative percentage plots for the residual scores for three OMIM gene lists. The OMIM gene categories are (a) autosomal dominant disease causing (N = 681), (b) autosomal recessive disease causing (N = 556), and (c) X-linked disease causing (N = 118). SORVA were based on the number of 1000 Genomes Project individuals who were heterozygous or homozygous for rare (MAF < 0.005) LOF variants in a given gene. Dashed lines indicate control. Abbreviations: ROC, Receiver Operating Characteristic; AUC, area under the curve, LOF, loss-of-function. (PDF 83 kb) [file 12920_2018_371_MOESM8_ESM.pdf]
