# Supplementary material for: Calculating the statistical significance of rare variants causal for Mendelian and complex disorders
Source: BMC Med Genomics. 2018 Jun 13;11:53. doi: 10.1186/s12920-018-0371-9 (PMC6001062; doi:10.1186/s12920-018-0371-9)
Supplement: Supplementary file 4 — Supplementary methods. Includes derivation of equations and math used for calculating the significance of finding rare variants in a given gene. (PDF 173 kb) [file 12920_2018_371_MOESM4_ESM.pdf]

Deriving the equation for calculating variant significance

1. Calculating significance of a *de novo* variant in a single-family study

Consider a case of a severe, pediatric-onset Mendelian disorder, in which both parents and the affected child are sequenced to identify the causal variant. If only *de novo* variants are identified within a putative gene, making a rough estimate of the *P*-value is relatively simple, as we need to calculate the probability of at least one *de novo* mutation occurring in a gene by random chance. The probability of two or more *de novo* mutations appearing in the same gene is close to zero and can thus be omitted. Ignoring variations in per-base mutation rates [1]:

$$P \approx l_{tx} d c$$

where l_{tx} is the length of the transcript in nucleotide bases and d is the mean rate of *de novo* single-nucleotide variants (SNVs) arising per nucleotide per generation. The genome-wide mutation rate is estimated to have a lower bound of 1.2×10^{-8} per site per generation [1–3], although sequencing technologies used in the studies are biased against GC-rich DNA, and the rate of mutation at CpG dinucleotides has been observed to be 10- to 18-fold the rate of non-CpG dinucleotides [4–7]. Therefore, the CpG content of a gene should also be considered when determining the parameter d. The parameter c is the fraction of *de novo* events that meet our protein consequence threshold. It is predicted that 2.85% of *de novo* events are splice site altering or nonsense events, and 70.64% of *de novo* events are protein-altering, i.e. missense or LOF [8]; these may be used as the respective values for c depending on the variant filtering criteria used.

If the sample size is one and all genes are considered equally likely to cause disease *a priori*, then the P-value may not be significant after correcting for the number of genes in the human genome; hence, follow-up studies are still required in such cases. To illustrate, a whole-exome sequencing study of a single pair of identical twins with autism and seizures identified a de novo missense variant in *KCND2* [9], which has a 5,331 base transcript, and the variant was confirmed by functional studies to support causality between the variant and phenotype. The uncorrected P-value would be calculated as $4.5 \times 10{-}5$, which is significant despite the small sample size. However, the P-value corrected for the number of sequenced genes—24,000 to be ultra-conservative—is not significant. In this case, the authors bolstered their study by performing functional studies. Generally, observing a *de novo* variant in an “N=1” study will not be significant, and the relative P-values of genes containing rare variation would be used to either prioritize genes to perform functional studies on, or to identify additional individuals with undiagnosed diseases who carry variants in the same gene, as previous studies have done [10, 11].

2. Observing homozygous variants in unrelated individuals

Let f_{hom} be the *a priori* fraction of individuals in the population that have a rare, homozygous variant in a given gene. Assume that we sequence n singletons and find that k of these individuals have a variant

in the gene. The random variable X is the number of times an individual is seen with a homozygous variant in the gene ("successes") out of n individuals sequenced ("independent trials"), and $X \sim \text{Binom}(n, \pi)$ where π is the parameter corresponding to the probability of success on any trial. Let H_0: $\pi \leq f_{hom}$ be the null hypothesis of no association between the phenotype and an individual being homozygous for a variant in the gene. Let H_1: $\pi > f_{hom}$ be the alternative hypothesis that we see a greater number of individuals with a homozygous variant in the gene than expected. The probability of getting exactly k successes is:

$$P(X = k) = \binom{n}{k} f_{hom}^{k} (1 - f_{hom})^{n-k} \ .$$

The one-sided p-value is the probability of observing at least k successes and can be expressed as:

$$P(X \geq k) = P(X > k-1) = 1 - P(X \leq k-1) = 1 - BinomCDF(k-1, n, f_{hom})$$

where BinomCDF denotes the binomial cumulative distribution function.

3. Calculating significance of observing heterozygous variants

Let's assume that we observe heterozygous variants in the gene of interest. A P-value is the probability of obtaining an effect at least as extreme as the one observed, assuming the truth of the null hypothesis. The effect that is at least as extreme as the one observed is equivalent to seeing at least as many individuals who are heterozygous *or homozygous* for a variant in the gene of interest. Therefore, the P-value becomes

$$P(X \geq k) = 1 - BinomCDF(k-1, n, f_{both})$$

where f_{both} is the fraction of individuals in the population that have either a heterozygous or homozygous variant in the gene of interest.

4. Sequencing multiple individuals within a family

If we sequence unrelated individuals, the probability that a single sequenced individual will have a homozygous or heterozygous variant in a given gene is f_{hom} or f_{both}, respectively, by definition. If we sequence related individuals, we can calculate the probability that the sequenced family members will share a homozygous or heterozygous variant within a gene using the Hardy-Weinberg equation. First, assuming a scenario where we only consider a single alternate allele existing within a gene, let's define:

$$f_{\text{hom}} = p^2$$
$$f_{het} = 2p(1-p)$$
$$f_{\text{hom}ref} = (1-p)^2$$

where p is the allele frequency for a rare variant.

4.1. Calculating the probability that full siblings share a homozygous variant within a gene or are both compound heterozygotes

If we sequence a sib pair, the probability that both siblings will share a homozygous variant can be derived from the probabilities of the parents having the following alleles:

Genotypes of mother and father:	Aa Aa	Aa AA<br>AA Aa	AA AA
Probability that child will be homozygous:	¼	½	1

Therefore, the probability that a sequenced sib pair will share a homozygous variant, for small values of *p*, can be calculated as:

$$P = (2p(1-p))^2 \times \tfrac{1}{4} \times \tfrac{1}{4} + 2(2p(1-p))p^2 \times \tfrac{1}{2} \times \tfrac{1}{2} + p^4$$
$$= \tfrac{1}{4} p^2 (1 - 2p + p^2) + (1-p)p^3 + p^4$$
$$= \tfrac{1}{4}(p^2 + 2p^3 + p^4)$$
$$\approx \tfrac{1}{4} p^2$$
$$= \tfrac{1}{4} f_{\text{hom}}$$

For small values of *p*, the probability that a parent is homozygous is negligible and can be ignored, and more simply, we can calculate:

$$P = (2p(1-p))^2 \times \tfrac{1}{4} \times \tfrac{1}{4}$$
$$= \tfrac{1}{4} p^2 (1 - 2p + p^2)$$
$$= \tfrac{1}{4}(p^2 - 2p^3 + p^4)$$
$$\approx \tfrac{1}{4} p^2$$
$$= \tfrac{1}{4} f_{\text{hom}}$$

If multiple loci are considered and we consider that the parents carry different heterozygous variants in the same gene, we redefine *p* as the sum of allele frequencies for rare variants within the gene. The assumption is that each allele is rare, and the frequency of individuals who are complex heterozygotes in the population is negligible. Then, the following approximation holds:

$$f_{\text{hom}} \approx p^2$$

For two parents whose haplotypes are Ab|ab and aB|ab, the probability of having a child who is compound heterozygous (Ab|aB) is ¼, and the probability of sibpairs sharing the compound heterozygous state can be calculated as previously. Since the variants are within the same gene, we may make the assumption that they are closely linked and recombination events between them are negligible.

If we sequence multiple sib pairs from independent families, let one sib pair consist a single trial, where the probability of success on any trial is $\tfrac{1}{4} f_{\text{hom}}$. Then, the one-sided *P*-value of observing shared

heterozygous variants in a given gene in k of n families becomes

$$P(X \geq k) = 1 - BinomCDF\left(k-1, n, \tfrac{1}{4} f_{\text{hom}}\right).$$

4.2. Calculating the probability that individuals in a consanguineous pedigree share a homozygous variant within a gene

Let's consider a more complicated case: determining the probability P that two sequenced individuals will share a homozygous variant in a family with consanguineous marriages. We assume that the sequenced individuals with a rare, autosomal recessive Mendelian disorder share the causal variant identical-by-descent (IBD). Then, we can calculate P based on the probability that the variant got passed from a common ancestor to each affected individual twice through independent paths. As an example, consider the following pedigree from Bilgüvar *et al.* (2010) [12]:

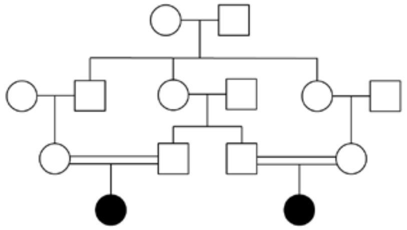

We can draw the following path diagram:

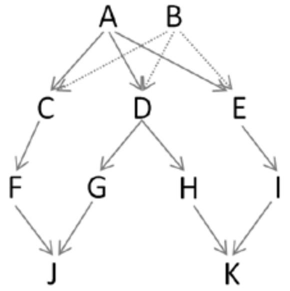

If one common ancestor, e.g. A, introduced a rare allele in a gene into the family with probability f_{het}, the only way both J and K could be homozygous for the variant is if the allele got passed down through each of the following 11 independent edges: $\overrightarrow{AC}, \overrightarrow{CF}, \overrightarrow{FJ}, \overrightarrow{AD}, \overrightarrow{DG}, \overrightarrow{GJ}, \overrightarrow{DH}, \overrightarrow{HK}, \overrightarrow{AE}, \overrightarrow{EI}$ and $\overrightarrow{IK}$ (solid lines in figure above). The probability of this happening through either common ancestor, because either A or B could have introduced the variant, can be calculated as:

$$P = 2 f_{het} \times \left(\tfrac{1}{2}\right)^{11}$$
$$= f_{het} \times \left(\tfrac{1}{2}\right)^{10} =$$
$$= f_{het} \times \left(\tfrac{1}{2}\right)^{E-1}$$

where E is the number of independent edges in the paths connecting the two sequenced individuals through a single common ancestor.

If we sequence pairs of individuals from multiple similar pedigrees, let one family consist a

single trial, where the probability of success on any trial is $\left(\frac{1}{2}\right)^{E-1} f_{het}$. Then, the one-sided *P*-value of observing shared homozygous variants in a given gene in *k* of *n* families becomes

$$P(X \geq k) = 1 - BinomCDF\left(k-1, n, \left(\tfrac{1}{2}\right)^{E-1} f_{het}\right).$$

4.3. Calculating the probability that multiple individuals in a family share a heterozygous variant within a gene

First, let's define the fraction of individuals in a population who are either heterozygous or homozygous for a rare variant in a gene as

$$f_{both} = f_{\mathrm{hom}} + f_{het} = p^2 + 2p(1-p) = 2p - p^2 \approx 2p$$

for small values of *p*, i.e. where $f_{both} << 1$. Note that in this case,

$$f_{het} = 2p(1-p) = 2p - 2p^2 \approx 2p$$
$$f_{het} \approx f_{both}$$

If we sequence a sib pair, the probability that both siblings will be heterozygous or homozygous for a variant in the gene can be derived from the probabilities of the parents having the following alleles:

Genotypes of mother and father:	Aa aa<br>aa Aa	Aa Aa	AA Aa<br>Aa AA	aa AA<br>AA aa	AA AA
Probability that child will be het or hom:	½	¾	1	1	1

Therefore, the probability that a sequenced sib pair will both be heterozygous or homozygous for a variant, for small values of *p*, can be calculated as:

$$P = 2(2p(1-p))(1-p)^2 \times \tfrac{1}{2} \times \tfrac{1}{2} + (2p(1-p))^2 \times \tfrac{3}{4} \times \tfrac{3}{4} + 2(2p(1-p)p^2 + 2p^2(1-p)^2 + p^4$$
$$= (1-p)^3 p + \tfrac{17}{4}(1-p)^2 p^2 + 4(1-p)p^3 + p^4$$
$$= p - p^4 + \tfrac{17}{4}p^2 - \tfrac{17}{4}p^4 + 4p^3 - 4p^4 + p^4$$
$$\approx p$$

For small values of *p*, we can assume that the sib pairs will share the variant IBD, and we can ignore the possibility that both parents were heterozygous or homozygous for variants in the gene. More simply, we can calculate:

$$P = 2(2p(1-p))(1-p)^2 \times \tfrac{1}{2} \times \tfrac{1}{2}$$
$$= (1-p)^3 p$$
$$= p - p^4$$
$$\approx p$$
$$= \tfrac{1}{2} f_{both}$$

We can generalize to more distantly related individuals, following the assumption that the heterozygous allele is shared IBD. The relationship coefficient *r*, which calculates the proportion of genes that two individuals have in common as a result of their genetic relationship, can be calculated as

$$r = \sum \left(\tfrac{1}{2}\right)^n$$

where *n* is the number of connecting links or paths separating the individuals in a path diagram [13]. Furthermore, if there is no inbreeding:

$$r = \left(\tfrac{1}{2}\right)^n + \left(\tfrac{1}{2}\right)^n = 2\left(\tfrac{1}{2}\right)^n = \left(\tfrac{1}{2}\right)^{n-1}$$

E.g. for full siblings *C* and *D*, the paths are $\overrightarrow{CAD}$ and $\overrightarrow{CBD}$:

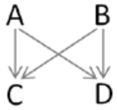

and $r = \left(\tfrac{1}{2}\right)^2 + \left(\tfrac{1}{2}\right)^2 = \tfrac{1}{2}$

If one common ancestor, e.g. *A*, introduced a rare allele in a gene into the family with probability f_{het}, the only way both descendants could be heterozygous for the variant is if the allele got passed down through each independent edge originating from *A*. The probability of this happening through either common ancestor, because either *A* or *B* could have introduced the variant, can be calculated as:

$$P = 2 f_{het} \times \left(\tfrac{1}{2}\right)^n$$
$$= \left(\tfrac{1}{2}\right)^{n-1} f_{het}$$
$$= rf_{het}$$
$$\approx rf_{both}$$

If we sequence pairs of individuals from multiple similar pedigrees, let one family consist a single trial, where the probability of success on any trial is rf_{both}. Then, the one-sided *P*-value of observing shared heterozygous variants in a given gene in *k* of *n* families becomes

$$P(X \geq k) = 1 - BinomCDF(k-1, n, rf_{both}).$$

5. Adjusting for cases where *f* = 0

When calculating the significance of observing homozygous or heterozygous variants in a gene for which no individuals have been observed with a rare variant in the population ($f_{hom} = 0$ or $f_{both} = 0$, respectively), then the calculated *P* value would mistakenly always seem significant. Therefore, for these cases, we set *f* to a very small number, arbitrarily to the pseudocount

$$f = \frac{1}{2N}$$

where *N* is the population size from which *f* was originally derived. The implicit assumption is that if we were to sequence twice the number of samples, we may observe a single individual with a variant in

the gene. For genes that are very rarely mutated, the value of *f* will still be overestimated, resulting in a conservative calculation of the *P*-value.

6. Combining P-values when families with multiple pedigree structures have been sequenced, with heterozygous variants within a given gene

Let's assume that we sequenced individuals in families with multiple family structures, e.g. we have sequenced independent cases and sib pairs with a rare, autosomal dominant Mendelian disorder, and we observed that *k* of *n* independent cases (singletons) and *j* of *m* sib pairs (doubletons) have heterozygous variants in a given gene. In this case, we have $n+m$ independent trials. The control population for calculating the likelihood of such an outcome is a pool of families such that the fraction of unrelated individuals versus sib pairs are equivalent to the fraction of unrelated individuals versus sib pairs sequenced, i.e. $\frac{n}{n+m}$ and $\frac{m}{n+m}$. In this control population, the fraction of unrelated individuals heterozygous for a variant in the gene is $f_{het} \approx f_{both} = r_0 f_{both}$ for $f_{het} << 1$ where the relationship coefficient is $r_0 = 1$, and the fraction of sib pairs who share heterozygous variants in the gene is $r_1 f_{both}$ where the relationship coefficient is $r_1 = ½$. Weighting these by the fraction of unrelated individuals and sib pairs, the total fraction of "familial units" that do not have or share the variant is

$$F = 1 - \tfrac{n}{n+m} r_0 f_{both} - \tfrac{m}{n+m} r_1 f_{both}$$

which equals the probability of a failure in any given trial. The probability $P_{n,m,k,j}$ of having $n+m$ trials and observing exactly k singleton successes and *j* doubleton successes is equal to the probability of observing exactly $n+m-k-j$ failures multiplied by the probability of observing exactly *k* successes out of $k+j$ trials:

$$P_{n,m,k,j} = P(X = n+m-k-j) \times P(Y = k)$$

where *X* is a binomial random variable with $n+m$ trials and probability of success equal to *F*, and *Y* is a binomial random variable with $k+j$ trials and probability of success equal to

$$\frac{\tfrac{n}{n+m} r_0 f_{both}}{\tfrac{n}{n+m} r_0 f_{both} + \tfrac{m}{n+m} r_1 f_{both}} = \frac{r_0 n}{r_0 n + r_1 m}$$

Therefore, the probability of observing the given combination of successful 'trials' can be calculated as

$$P_{n,m,k,j} = \binom{n+m}{n+m-k-j} F^{n+m-k-j} (1-F)^{k+j} \times \binom{k+j}{k} \left(\frac{r_0 n}{r_1 m}\right)^k \left(1 - \frac{r_0 n}{r_1 m}\right)^j$$

$$= \binom{n+m}{n+m-k-j} (1 - \tfrac{n}{n+m} r_0 f_{both} - \tfrac{m}{n+m} r_1 f_{both})^{n+m-k-j} (\tfrac{n}{n+m} r_0 f_{both} + \tfrac{m}{n+m} r_1 f_{both})^{k+j} \times \binom{k+j}{k} \left(\frac{r_0 n}{r_1 m}\right)^k \left(1 - \frac{r_0 n}{r_1 m}\right)^j$$

Finally, to calculate the P-value for observing *k* of *n* independent cases and *j* of *m* sib pairs who have

heterozygous variants in a given gene, we calculate the probability $P_{n,m,k,j}$ of observing exactly *k* singleton successes and *j* doubleton successes or any combination of outcomes that is less likely, and sum these values.

$$P - value = \sum_{a=0}^{n}\sum_{b=0}^{m} P_{n,m,a,b}\left[P_{n,m,a,b} \leq P_{n,m,k,j}\right]$$

The *P*-value can be derived in a similar manner for various experimental designs, where multiple families with different pedigree structures are sequenced to identify heterozygous variants shared by affected cases or, in case of an autosomal recessive disorder, homozygous or potential compound heterozygous variants.

References

1. Campbell CD, Eichler EE: Properties and rates of germline mutations in humans. *Trends Genet TIG* 2013, **29**:575–584.

2. Veltman JA, Brunner HG: De novo mutations in human genetic disease. *Nat Rev Genet* 2012, **13**:565–575.

3. Conrad DF, Keebler JEM, DePristo MA, Lindsay SJ, Zhang Y, Casals F, Idaghdour Y, Hartl CL, Torroja C, Garimella KV, Zilversmit M, Cartwright R, Rouleau GA, Daly M, Stone EA, Hurles ME, Awadalla P, 1000 Genomes Project: Variation in genome-wide mutation rates within and between human families. *Nat Genet* 2011, **43**:712–714.

4. Kondrashov AS: Direct estimates of human per nucleotide mutation rates at 20 loci causing Mendelian diseases. *Hum Mutat* 2003, **21**:12–27.

5. Kong A, Frigge ML, Masson G, Besenbacher S, Sulem P, Magnusson G, Gudjonsson SA, Sigurdsson A, Jonasdottir A, Jonasdottir A, Wong WSW, Sigurdsson G, Walters GB, Steinberg S, Helgason H, Thorleifsson G, Gudbjartsson DF, Helgason A, Magnusson OT, Thorsteinsdottir U, Stefansson K: Rate of *de novo* mutations and the importance of father's age to disease risk. *Nature* 2012, **488**:471–475.

6. Campbell CD, Chong JX, Malig M, Ko A, Dumont BL, Han L, Vives L, O'Roak BJ, Sudmant PH, Shendure J, Abney M, Ober C, Eichler EE: Estimating the human mutation rate using autozygosity in a founder population. *Nat Genet* 2012, **44**:1277–1281.

7. Lynch M: Rate, molecular spectrum, and consequences of human mutation. *Proc Natl Acad Sci* 2010, **107**:961–968.

8. Kryukov GV, Pennacchio LA, Sunyaev SR: Most rare missense alleles are deleterious in humans: implications for complex disease and association studies. *Am J Hum Genet* 2007, **80**:727–739.

9. Lee H, Lin MA, Kornblum HI, Papazian DM, Nelson SF: Exome sequencing identifies de novo gain of function missense mutation in KCND2 in identical twins with autism and seizures that slows potassium channel inactivation. *Hum Mol Genet* 2014, **23**:3481–3489.

10. Chong JX, Yu J-H, Lorentzen P, Park KM, Jamal SM, Tabor HK, Rauch A, Saenz MS, Boltshauser E, Patterson KE, Nickerson DA, Bamshad MJ, Genomics U of WC for M: Gene discovery for Mendelian conditions via social networking: de novo variants in KDM1A cause developmental delay and distinctive facial features. *Genet Med* 2016, **18**:788–795.

11. Takenouchi T, Miura K, Uehara T, Mizuno S, Kosaki K: Establishing SON in 21q22.11 as a cause a new syndromic form of intellectual disability: Possible contribution to Braddock–Carey syndrome phenotype. *Am J Med Genet A* 2016, **170**(10):2587–90.

12. Bilgüvar K, Öztürk AK, Louvi A, Kwan KY, Choi M, Tatli B, Yalnizoğlu D, Tüysüz B, Çağlayan AO, Gökben S, Kaymakçalan H, Barak T, Bakircioğlu M, Yasuno K, Ho W, Sanders S, *et al.*: Whole exome sequencing identifies recessive *WDR62* mutations in severe brain malformations. *Nature* 2010, **467**(7312), 207–210.

13. Wright S: Coefficients of inbreeding and relationship. The American Naturalist 1922, **56**(645), 330–338.
